# Complete mitochondrial sequences from Mesolithic Sardinia

**DOI:** 10.1038/srep42869

**Published:** 2017-03-03

**Authors:** Alessandra Modi, Francesca Tassi, Roberta Rosa Susca, Stefania Vai, Ermanno Rizzi, Gianluca De Bellis, Carlo Lugliè, Gloria Gonzalez Fortes, Martina Lari, Guido Barbujani, David Caramelli, Silvia Ghirotto

**Affiliations:** 1 Dipartimento di Biologia, Università di Firenze, 50122 Florence, Italy; 2 Dipartimento di Scienze della Vita e Biotecnologie, Università di Ferrara, 44121 Ferrara, Italy; 3 Fondazione Telethon, 20121 Milano, Italy; 4 Istituto di Tecnologie Biomediche, CNR, 20090 Segrate, Milano, Italy; 5 LASP, Dipartimento di Storia, Beni Culturali e Territorio, Università di Cagliari, 09124 Cagliari, Italy

## Abstract

Little is known about the genetic prehistory of Sardinia because of the scarcity of pre-Neolithic human remains. From a genetic perspective, modern Sardinians are known as genetic outliers in Europe, showing unusually high levels of internal diversity and a close relationship to early European Neolithic farmers. However, how far this peculiar genetic structure extends and how it originated was to date impossible to test. Here we present the first and oldest complete mitochondrial sequences from Sardinia, dated back to 10,000 yBP. These two individuals, while confirming a Mesolithic occupation of the island, belong to rare mtDNA lineages, which have never been found before in Mesolithic samples and that are currently present at low frequencies not only in Sardinia, but in the whole Europe. Preliminary Approximate Bayesian Computations, restricted by biased reference samples for Mesolithic Sardinia (the two typed samples) and Neolithic Europe (limited to central and north European sequences), suggest that the first inhabitants of the island have had a small or negligible contribution to the present-day Sardinian population, which mainly derives its genetic diversity from continental migration into the island by Neolithic times.

Due to its geographic isolation in the Mediterranean sea, the biological history of Sardinia has been the subject of extensive anthropological and population-genetics investigation. Several studies based on autosomal markers^1^,^2^,^3^,^4^, mitochondrial DNA (mtDNA)^5^,^6^,^7^,^8^,^9^ and Y-chromosome polymorphisms^10^,^11^,^12^,^13^ showed that the Sardinian population is one of the main European genetic outliers^14^,^15^,^16^,^17^ and reported unusually high levels of internal diversity^18^,^19^. Most of these studies compared variation in Sardinia and in other European populations, but there is still uncertainty about past population dynamics and demographic processes within the island, as well as about the exact nature and the extent of the genetic exchanges that occurred over millennia, actually determining the existing Sardinian genetic structure.

Controversy has also surrounded the origins and the antiquity of the colonization of Sardinia. The earliest presence of humans is still under debate. Some authors likely date it back to the end of the Middle Pleistocene, on the base of lithic artifacts typology, attributed to the Lower Paleolithic^20^,^21^,^22^. Nonetheless, neither human remains nor absolute-dated contextual evidence support this hypothesis. However, clues of human settlements arose only from the end of the Upper Pleistocene^23^,^24^, with single human remains discovered out of context and dated back to 20,000 years ago just on the base of stratigraphic correlations^25^. The first evidence of Holocene frequentation of the island are scattered in a few rock-shelters and caves, exclusively on the inside of a 20 km coast belt^26^. After this poorly-documented phase, with around 500 years hiatus of archaeological evidence, with the advent of the agriculture, the population of the island increased in size, as demonstrated by the density of Early Neolithic (EN) sites (VI millennium BCE), and at the beginning of the IV millennium BCE, starting from the Final Neolithic culture of *Ozieri*, there has been a rapid growth of archaeological documentation and skeletal remains^27^,^28^,^29^,^30^. The fragmented anthropological and archaeological evidence of the Pre-Neolithic phase make it difficult to properly describe a continuity towards the process of Neolithization in Sardinia; however, the gap in the archaeological findings of the two periods suggests a lack of interaction between Mesolithic and EN groups.

From a genetic perspective, a recent genomic study of both ancient and modern Europeans, including data from more than 400 modern-day Sardinians, revealed the existence of genetic affinities between Neolithic Europeans samples and modern Sardinians. According to the authors, these results not only indicate a Neolithic origin of modern Sardinians, but also suggest that Sardinians are a “modern-day ‘snapshot’ of the genetic structure of the first farmers associated with the spread of agriculture in Europe”^31^. Unfortunately, this hypothesis has not been supported so far by evidence coming from ancient Sardinian genetic data, due to the paucity of Pre-Neolithic and actual absence of EN human remains. The only ancient data ever published were sequences of the mtDNA control region from Bronze-Age sample^8^ and revealed a directed genealogical continuity between Nuragic individuals and the current people of Ogliastra, but not of Gallura^9^. Past dispersal dynamics, genetic exchanges and replacements during the Neolithic in continental Europe have been extensively studied by means of ancient genetic data^32^,^33^,^34^,^35^,^36^,^37^,^38^,^39^,^40^,^41^,^42^; although the general European picture is getting clearer, many aspects of the Neolithic transition in Sardinia are still poorly understood, starting from whether, and to what extent, gene flow from mainland Europe during the time of the spread of agriculture actually contributed in shaping the genetic makeup of the island.

With this study, we present the first two complete mitochondrial genome sequences of Mesolithic human remains from Sardinia, dated back to around 10,000 yBP and associated with the earliest direct evidence of human presence in the island^43^. We analyzed these sequences along with modern and ancient genetic data in order to contextualize the Mesolithic Sardinian haplotypes into the European genetic variation, as well as to investigate the Paleolithic contribution to the current Sardinian gene pool. Preliminary model testing under an Approximate Bayesian Computation (ABC) framework is so far, given the extremely limited reference samples for Mesolithic Sardinia and Neolithic Europe supporting the hypothesis that modern-day Sardinian genetic variation is mostly derived from a massive migration from continental Europe during Neolithic times.

## Results

### Samples and sequencing

**Figure 1:**
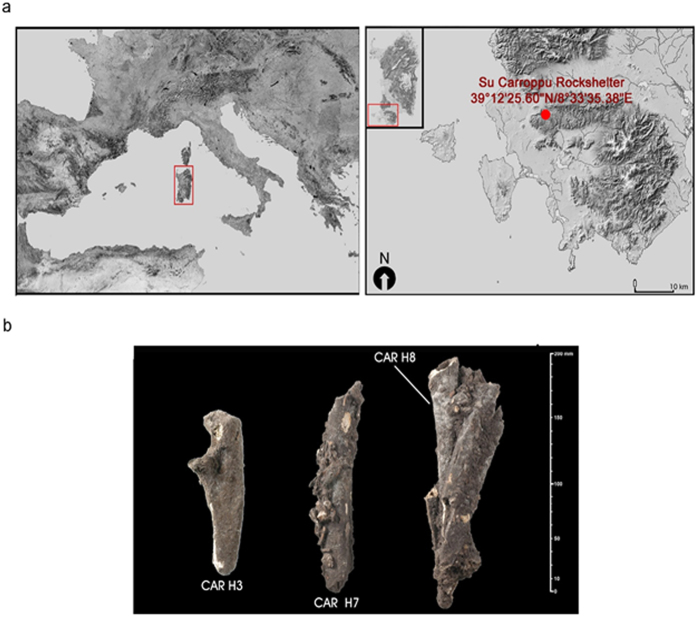
Su Carroppu site and samples. (**a**) the location of Su Carroppu rockshelter, Sardiania (Italy) and (**b**) pictures of the 3 samples used in this study. The map is plotted using data available on http://webgis.regione.sardegna.it/Download/raccolteCartografiche/modelliDigitaliTerreno/DTM10m/.The material is licensed under the Creative Commons attribution 4.0 International license (https://creativecommons.org/licenses/by/4.0/legalcode). The map was processed with Corel Photo-Paint 9 v9.439 (http://www.coreldraw.com/en/product/graphic-design-software/? topNav=en, version 9.439 licensed to CL) and modified with Photoshop CC (2015.5).

We analyzed the remains of three individuals excavated from the Su Carroppu rockshelter of the Sulcis region (Fig. 1, Supplementary Fig. S1a and b). The Su Carroppu site plays a relevant role in Sardinia, with a remarkably rich archaeological record and a series of occupational phases spanning from the Mesolithic to the historical period. The 1978 archaeological excavations in the lowermost layer (level-4) (Supplementary Fig. S1c), yielded a large quantities of remains, including fragments of human bones intermingled with bones of *Prolagus sardus*. Three direct radiocarbon dates performed on the human bones placed the remains in the mid-9^th^ millennium cal. BCE (Table 1; Supplementary Table S1) thus showing an unexpected Early Mesolithic settlement predating EN occupation^43^,^44^.

Here we reconstructed nearly complete mitochondrial genomes for two individuals from Su Carroppu (CAR-H7 and CAR-H8, Table 1), using hybridization capture in solution^45^ coupled with high-throughput sequencing. A third individual from the same site (CAR-H3) was also captured and sequenced, but the resulted sequences did not reached the standard quality requested to guaranty the reliability of the NGS data and the sample was excluded for further analysis. The samples displayed typical features of aDNA^46^: short fragments, with average length <65 base pairs (bp), and high rate of cytosine deamination at the 5′ end of the molecules (Table 1; Supplementary Fig. S2; Supplementary Table S2). To further assess authenticity in our ancient mitochondrial genomes we evaluated the percentage of possible contaminant reads by estimating the amount of secondary bases at each haplogroup-defining positions: excluding the putative damaged bases, CAR-H7 reached the 4.04% and CAR-H8 reached the 3.38% (details in Supplementary Table S3), values that are within the range of expected contaminants considering the observed figures for published aDNA mitogenomes^33^,^40^. We also computed Bayesian contamination estimate^47^: the contamination ranging between 0.9–7.3% for CAR-H7 and 0.4–5.9% for CAR-H8 and the probability of authenticity was high in all the two samples, i.e. 0.95 for CAR-H7 and 0.98 for CAR-H8 (Table 1; Supplementary Fig. S3; Supplementary Table S2). The mitochondrial haplogroups were called using HaploGrep^48^,^49^ (Table 1, Supplementary Table S4); the diagnostic variants showed a coverage ranging from 5 to 28 and were further verified by visual inspection. The CAR-H8 sample belongs to haplogroup I3, hence representing, to the best of our knowledge, the first pre-Neolithic sample carrying the haplogroup I. Studies based on complete mitogenomes have previously reported haplogroup I in ancient samples from Iran (individual I674, haplogroup I1c) and Levant (individual I1679, haplogroup I), dated to 5,105 ± 35 yBP and 8,850–8,750 yBP, respectively^39^. It was also found in two late Neolithic individuals from Germany, both belonging to haplogroup I3a and dated to around 4,000 yBP^50^ but not in previous periods in Europe. Nowadays, this haplogroup is uncommon; its frequency is about 2% in modern Sardinians, 3% across Europe, and raises at maximum 6% in Northern European countries^51^. This is the first time that haplogroup I is found in a Mesolithic individual in Europe and the fact that we recovered this haplogroup in a sample of only two sequences may mean that it was present at higher frequencies in pre-Neolithic Sardinians or, in general, in the population that first settled in the island. The other sample (CAR-H7) belongs to the haplogroup J2b1. The haplogroup J has already been found in late hunter-gatherer European populations, with a frequency of about 4%^32^. The current frequency of the haplogroup J is higher than that of the haplogroup I, variable in Europe from 1.7% (Caucasus) to 15% (Wales), and representing the 13% of the total modern Sardinians mitochondrial sequences.

### Network analyses

**Figure 2:**
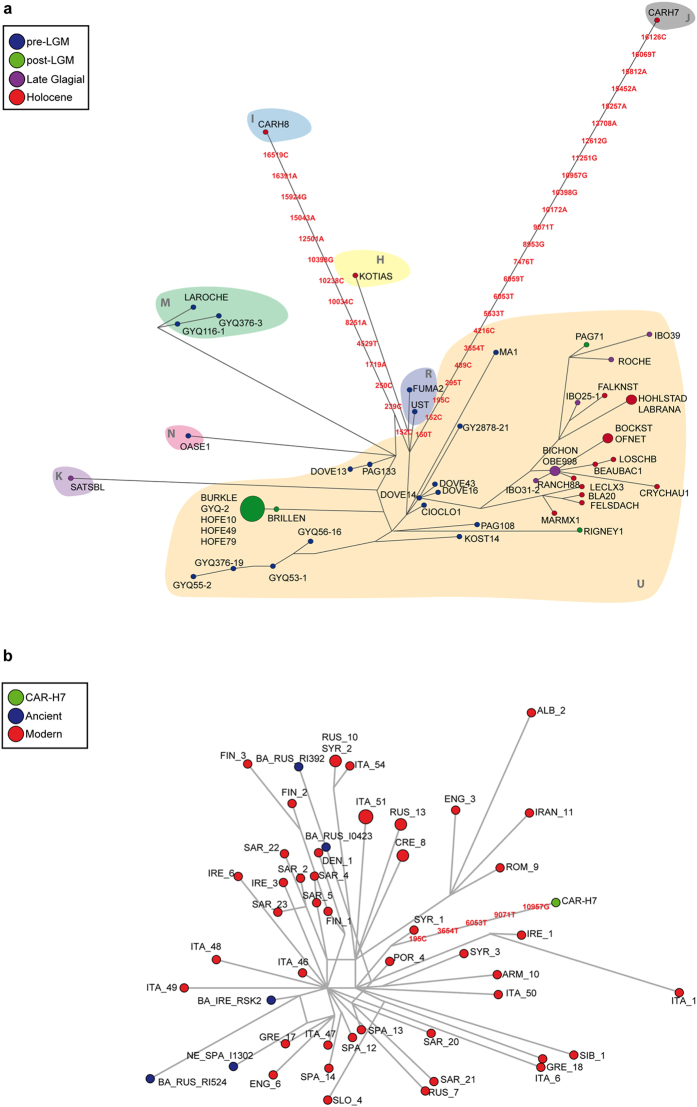
Median-joining network based on nucleotide variation in the whole mtDNA within (**a**) Pre-Neolithic dataset (Supplementary Table S5) (**b**) J2b dataset (Supplementary Table S6).

We performed a median-joining network analysis^52^ to determine the phylogenetic position of the two newly-discovered sequences within the context of the genetic diversity among Pre-Neolithic complete sequences (Supplementary Table S5). Despite the network (Fig. 2a) shows a temporal pattern from left (pre-LGM) to right (Holocene), the Sardinian sequences occupy a peculiar position, not together with coeval sequences (red circles). The background shading indicates the affiliation of the lineages to the major haplogroup definition (that were determined with HaploGrep^48^ based on PhyloTree Build 16). Among the non-Sardinian Pre-Neolithic samples, the most frequent major haplogroup is U, represented by 41 sequences. Just a few more haplogroups are present, namely H, K, M (three sequences each), N, R (two sequences each). The two Sardinian haplogroups (I3 and J2b1) appear well differentiated from each other and from all the other haplogroups considered in the analysis.

The Mesolithic CAR-H7 sample represents so far the oldest sequence belonging to haplogroup J2b. To better investigate the phylogeographic variation of this sequence respect to other European and Sardinian sequences belonging to the same haplogroup, we collected a dataset with 48 modern and 5 ancient J2b sequences (Supplementary Table S6) and we performed a median-joining network analysis^52^. The network confirmed that the sample CAR-H7 (green dot) falls within the variation expected for the haplogroup J2b, although carrying 5 private polymorphisms (195C, 3654T, 6053T, 9071T, 10957G) (Fig. 2b). The modern Sardinian J2b haplotypes seem to be well differentiated from the Mesolithic sequence. (Fig. 2b).

### Sardinian past demographic history

**Figure 3:**
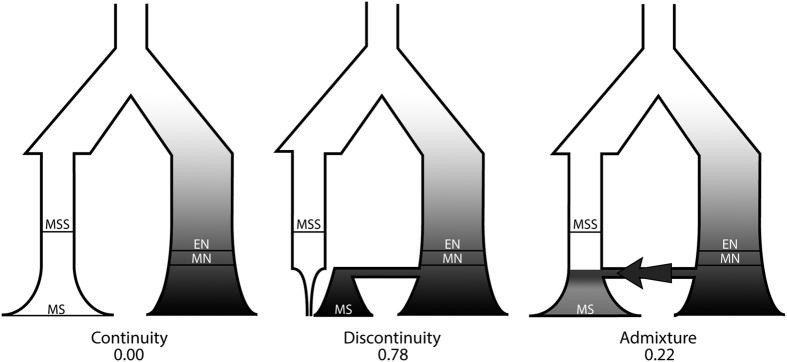
Alternative models of the genealogical relationships among past and present populations, and their posterior probabilities based on 50,000 best fitting simulations. MSS: Mesolithics from Sardinia; MS: Moderns from Sardinia; EN: Early Neolithics; MN: Middle Neolithics.

We then investigated the past demographic history and the genealogical relationships through 10,000 years in Sardinia by Approximate Bayesian Computation^53^,^54^. We first defined three alternative models of evolution, shown in Fig. 3. The first, which we called “*continuity*”, assumed modern inhabitants of Sardinia to be direct descendants of a Mesolithic Sardinian population, without any genetic exchange with continental Europe. The second, which we called “*discontinuity*”, assumed a complete replacement of ancient Mesolithic Sardinia by Neolithic people from Continental Europe. Under the third model the current inhabitants of Sardinia are a genetic mixture of local Mesolithic individuals and Neolithic individuals from the continent. We called this model “*admixture*”. We performed 500,000 coalescent simulations under each model, with parameter values randomly chosen from prior distributions (see Supplementary Materials for details). We calculated the models’ posterior probabilities by weighted multinomial logistic regression^53^, evaluating different thresholds to check the stability of the results. As it is shown in Fig. 3 and in Supplementary Table S7 the *continuity* model received essentially no support, with the *discontinuity* model having the highest probability (78%). The *admixture* model received poor support (22%), with the best fit obtained when 75% of current inhabitant of Sardinia come from a continental Neolithic population (modal value, see Supplementary Fig. S4 and Supplementary Table S8), that is on the upper bound of its prior distribution.

We determined the accuracy of our model choice inference by calculating the true and the false positive rates using 1,000 random simulations from each model as pseudo-observed datasets; the results are shown in Supplementary Table S9. The true positives rate was high for all the models, ranging from 0.64 to 0.89. The false positive rate was below 0.05 for the *discontinuity* model, and relatively low, but higher, for the *continuity* and the *admixture* model (0.084 and 0.157 respectively). In general, these results mean that the model we tested can be well recognized by the model selection procedure we adopted. We also evaluated the fit of the *discontinuity* model calculating a p-value for the observed dataset under an estimated general linear model (as in Wegmann *et al*.^55^), which can also be used to judge if the observed data are in agreement with the data simulated. The so calculated p-value was not significant (0.57), meaning that the observed data are plausible under the model we selected as the best one. The Principal Component Analyses of the best 5,000 simulations coming from each model actually confirmed that the *discontinuity* model is able to generate the observed variation, and that only a poorer fit is given by the *admixture* and the *continuity* model (Supplementary Fig. S5).

To better understand the role of the Neolithic migration in shaping the current Sardinians mitogenome variation we then simulated an admixture model (that we called “*admixture_tot*”) in which the proportion of lineages of current Sardinians coming from the Neolithic Europe was free to vary from 0 (complete continuity with Mesolithic in Sardinia) to 1 (complete replacement of Sardinian Mesolithics). We estimated the demographic parameters of this model (Table 2 and Supplementary Fig. S6), using summary statistic transformed via Partial Least Square^56^ (see Supplementary Materials for details). All the parameters resulted to be well estimated, as it is shown by their R squared values, in some cases higher than 0.6. The median and the mode values of the proportion of Neolithic mitochondrial lineages that gave rise to current Sardinians were 0.87, and 0.96 respectively, implying that a large proportion of the current mtDNA variation in Sardinia does not come from the first inhabitants of the island. We estimated these first incomers having an effective population size of about 790 individuals, with a 95% HPD ranging between 100 and 2,700 individuals. Current Sardinian effective population size was estimated to be predictably higher, with a median value of about 21,000 individuals and a wider 95% HPD. The median value of the mutation rate was estimated to be 2.1*10–8 mutations per nucleotide per year, considering a generation time of 30 years^57^, that is almost identical to the value estimated by Fu *et al*.^47^

## Discussion

Archaeological evidence suggests the first human presence in Sardinia around 20,000 years ago^25^, with sporadic and discontinuous occupations during Paleolithic and Mesolithic ages. Nowadays, Sardinians form a distinct outlier within the genetic variation of modern Europeans^14^, often interpreted as a consequence of thousands years of genetic isolation and drift, but little is known about the demographic changes that could have shaped the observed pattern of genetic variation. The, so far limited, ancient Sardinia genetic data allowed us to highlight a complete genetic continuity within a specific region of the island, Ogliastra, since the Bronze-Age^9^; however, cranial morphological evidence suggests that this continuity may have been established since Neolithic times, and possibly earlier^58^.

The two Mesolithic sequences retrieved in the Su Carroppu archaeological site represent the oldest sample of DNA in Sardinia, thus providing a direct genetic evidence about the first colonizers of the island. The samples were treated following all the golden criteria before DNA extraction and sequencing to avoid any contamination. To determine the mitochondrial haplogroups, trimmed reads were mapped against the reference sequence and only high quality calls, with a quality score of 30 or more were kept (detailed in Supplementary Materials). The comparable mitochondrial DNA data from European late hunter-gatherers have shown a remarkable genetic uniformity among pre-Neolithic populations, with most of the sequences (∼83%) belonging to the haplogroup U, of which a majority carry U5 haplotypes (>65%)^32^. Neither Sardinian sequence belongs to any of the U haplotypes, documenting the presence of substantial genetic differences over the Mediterranean area. In addition, neither sequence has been observed in later, ancient or contemporary, individuals, and both belong to haplogroups and subhaplogroups now present in Europe at low (J,<16%) or very low (I,<7%) frequencies, and that are rare in modern Sardinia. Based on complete mitochondrial genomes, Posth *et al*.^41^ described a higher genetic diversity in pre-LGM than in post-LGM European populations and identified a major turnover around 14,000 yBP, with the subsequent expansion of haplogroup U that became widespread all around Europe until the Neolithic transition. We do not find haplogroup U in Sardinia by 11,000 yBP, which means a different impact of the LGM in the island, and probably a high isolation of the Sardinian population, not only from Neolithic times onwards (as genomes data seems to have probed), but also from former times considering the dates of our samples.

The phylogenetic network analysis of all the Pre-Neolithic complete mitochondrial sequences so far generated, actually confirmed this view (Fig. 2a). The majority of the Late Pleistocene and Early Holocene sequences belongs to the U lineage, and form a quite homogeneous cluster at the bottom of the network. The two Mesolithic samples from Sardinia are highly differentiated, departing from the network through long branches, so as to indicate mutations possibly arising along thousand years of geographic (and genetic) isolation. The genome-wide data of Ice Age hunter-gatherers have shown that prehistoric Europe was characterized by recurrent populations turnover and migrations^42^, which resulted in a genetic homogeneity across pre-Neolithic populations. So far, our two ancient Sardinian sequences seem to support the view that these ancient populations movements did not involve genetic exchanges with Sardinians: isolation and drift may have resulted in a substantial mitochondrial differentiation between them and other Europeans. A larger characterisation of ancient sequences across the Mediterranean will help to clarify this suggestion.

The role and the genetic impact of migrations in Sardinia from continental Europe has been under debate for years^9^,^10^,^31^, with particular interest on whether, and to what extent, the gene flow from the mainland during the time of the spread of agriculture in Europe contributed to shaping the present Sardinian gene pool^31^. We then explicitly compared demographic models through Approximate Bayesian Computation^53^,^59^. The question thus addressed was not whether the two Su Carroppu Mesolithic individuals are ancestral to current Sardinians along the maternal line (of course, they are not), but rather what was the posterior probability that a population of size 100–10,000 individuals (the broad interval of priors considered), and comprising the Su Carroppu individuals, may have contributed to the current gene pool. Because the alternative to genealogical continuity since Mesolithic times is immigration from the mainland, Middle and Early Neolithic sequences from Central Europe^50^ were included as a source of Neolithic migrants into the island. This is not the best reference panel but we were limited to use it given the lack of Neolithic sequences from South Europe. Results must be interpreted with caution. A model of genealogical continuity in Sardinia since Mesolithic times appeared very unlikely. We could not discriminate between a model assuming a certain degree of admixture and one of complete replacement by Neolithic immigrants, but if admixture occurred the contribution of Mesolithic people was apparently very limited (Fig. 3).

We assessed the quality of these results by a number of tests. First, we evaluated exactly the probability to obtain false positives in the estimation of models’ posterior probabilities, with the discontinuity model having the lowest type one error. Then, we showed in various ways (posterior predictive p-value and PCA analysis) that the discontinuity model can in fact reasonably reproduce the observed variation. Clearly, a certain degree of uncertainty necessarily affects any analysis, particularly when it is based on a single DNA region and on the necessarily small samples in which ancient DNA is usually typed.

When explicitly estimating the Neolithic admixture proportion, i.e. the amount of Neolithic genes from continental Europe that gave rise to the current Sardinian genetic pool (*admixture_tot* model), we obtained values of 0.8–0.9%, depending on the point estimates considered (Table 2). This means that a significant proportion of modern Sardinian mitochondrial variation would came not from its first settlers, but from a subsequent migration wave from the continent. These results need to be tested in the future when reference ancient datasets are extended for both Mesolithic Sardinia and Neolithic Mediterranean. It is well accepted in the literature that the Neolithization of Europe proceeded in two waves, one for Central and North Europe, and the other for South Europe/Mediterranean^33^,^35^,^60^,^61^. But currently, there is no good proxy available for the ancient Neolithic Mediterranean pool. In our model comparison, we fixed the time of this second migration to 6,000 years ago, thus compatible with the archaeological evidence of Neolithic expansion in Sardinia. The spread of agriculture in Sardinia would hence been associated with demic diffusion from the continent, resulting in a large-scale population replacement. These results, for the first time supported by ancient genetic data, are also in good agreement with archaeological evidence and with what emerged from the comparison of modern Sardinian genomic data and Neolithic and Paleolithic sequences^31^, interpreted by the authors as evidence of gene flow from mainland Europe during the time of the spread of agriculture in Europe. Sikora *et al*.^31^ also envisaged a genetic continuity until present times, but did not provide quantitative measures of it. Another possibility, compatible with our results, would be that Sardinian Paleo-Mesolithic males, but not females, admixed with immigrants from Neolithic Europe. This, however, would mean that in Sardinia the spread of the Neolithic culture was mainly carried out by women, in contrast with the available evidence^62^. Moreover, this view is also in contrast with studies of sex-biased admixture in modern communities, suggesting that the invading population tends to incorporate female residents more than males^63^,^64^,^65^,^66^,^67^.

In conclusion, this study, albeit limited to DNA transmitted along the female lines of descent, provides the first genetic evidence on the earliest inhabitants of Sardinia, who bear maternal lineages distinct from current ones. Based on these two sequences, it seems that the Neolithization of the island was not a local development, but was associated with the arrival of a genetically-distinct group of immigrants from continental Europe.

## Methods

### DNA extraction and Sequencing

All extraction and library preparation steps before amplification were performed in the clean-room facilities of the Laboratory of Molecular Anthropology and Paleogenetics, University of Florence. Preventive measures were taken to avoid contamination during all experiments.

Sample surface was mechanically removed using a dental micro-drill with disposable tools, then the samples were UV-irradiated (254 nm) for 1 hour. Samples were ground to fine powder using the same dental micro-drill at very slow rotation (1000 rpm) and stored at −20 °C until further use. For each sample, DNA was extracted from 100 mg of bone powder following a silica-based protocol^68^. A 25 μl aliquot of each extract was used to produce double-stranded and double-indexed libraries according to a modified Illumina multiplex protocol^69^. All libraries were amplified to reach plateau and enriched for human mtDNA in a bead-capture method using long-range PCR products as bait for hybridization^45^. Negative controls were processed during each experimental step (see Supplementary Materials and Supplementary Table S10 for details).

Enriched libraries were pooled in equimolar amount with libraries from other samples and sequenced in paired-end (2 × 75 + 8 + 8 cycles) on the Illumina MiSeq platform at the Institute of Biomedical Technologies, National Research Council, in Segrate (Milano).

### NGS Data Processing and Authentication

Paired-end reads were merged into single reads and the adapters were trimmed using SeqPrep^70^. Filtered reads were mapped against the revised Cambridge Reference Sequence (rCRS) using BWA^71^, setting “-l 1000 -n 0.01 -o 2” optimized for increased sensitivity for aDNA; reads with mapping quality below 30 were discarded and PCR duplicates were collapsed into consensus sequences. To estimate the misincorporation pattern at the end of the reads, BAM files were run on *mapDamage2.0*^72^. Then, to test for the authenticity of the consensus sequences, we used a Bayesian contamination estimate to calculate the probability that the recovered mtDNA fragments come from a single biological source^47^. A detailed description can be found in Supplementary Materials, and in Supplementary Table S11.

### Haplogroup identification

Consensus sequences were called using samtools packages^73^: only high quality calls with a quality score of 30 or more were kept. The two sequences were uploaded on HaploGrep^48^,^49^ to assign the mitochondrial genome to known haplogroups and call mtDNA SNPs, followed by manual verification of each diagnostic variant.

In order to reduce the loss of the information, the assemblies were subsequently visually inspected.

### Network analysis

The phylogenetic networks based on nucleotide variation in the whole mtDNA, were constructed using the Median Joining algorithm^52^ implemented in Network 5.0 program (http://www.fluxus-technology.com). The ε value was set to 0 and the transversions were weighted 3x the weight of transitions. Networks were subjected to maximum parsimony post-analysis.

### Approximate Bayesian Computation

We implemented the ABC framework using the *ABCsampler* tool in the ABCToolbox package^55^. We simulated genetic data under three demographic models (*continuity, discontinuity* and *admixture*, see Fig. 3, detailed in Supplementary Materials) with *fastsimcoal2* (ver 2.5.2.21)^74^ and running 500,000 simulations per model. The prior distributions we considered are detailed in Supplementary Table S12. The modern Sardinian sample includes 63 sequences from Ogliastra^75^, the unique unbiased sample of Sardinian complete mitochondrial genomes available. As source of Neolithic variation we used 18 Middle Neolithic (6,500–5,000 BCE) and 28 Early Neolithic (7,300–6,200 BCE) sequences from Haak *et al*.^50^, that are the Early and Middle Neolithic samples with the highest quality (see Supplementary Materials and Supplementary Table S13). We placed ancient samples in the corresponding branch of the demographic model, at an average sampling time. To compare models we applied the Logistic Regression procedure^59^, considering different thresholds (i.e. number of retained simulations) to check the consistence oh the results. Model parameters were estimated by a locally weighted multivariate regression^53^ after a *logtan* transformation^76^ of the 5,000 best-fitting simulations from a specific model. To calculate the posterior probabilities for models and parameters we used R^77^ scripts from http://code.google.com/p/popabc/source/browse/#svn%2Ftrunk%2Fscripts, modified by SG. We also estimated the power of our ABC procedure to correctly recognize the true model calculating for each model the proportion of true positives and false positives. We evaluated 1,000 random pseudo-observed data sets generated under each model, counting the number of times a specific model is correctly identified by the ABC procedure (true positives), and the number of times the same model is incorrectly selected as the true model (false positives). The PCA was made with the *PCA* function of the *FactoMineR* package^77^,^78^.

## Additional Information

**Accession Codes:** The accession numbers for the two mtDNA genome sequences reported in this paper are GenBank: KX354973-KX354974.

**How to cite this article:** Modi, A. *et al*. Complete mitochondrial sequences from Mesolithic Sardinia. *Sci. Rep.* **7**, 42869; doi: 10.1038/srep42869 (2017).

**Publisher's note:** Springer Nature remains neutral with regard to jurisdictional claims in published maps and institutional affiliations.

## Supplementary Material

Supplementary Material

## Figures and Tables

**Table 1:** Samples analyzed.

Sample ID	^14^C Age (BCE)	nt covered at least at 3-fold coverage (% of mtDNA)	Average fragment lenght	C to T misincorporation at 5′-end (%)	Contamination estimate (95% CI)	Hg
CAR-H3	7938–7525	13,730 (82.88%)	72.74	N/A	N/A	N/A
CAR-H7	8227–7596	16,527 (99.75%)	62.59	34.45	0.9–7.3%	J2b1
CAR-H8	9124–7851	16,446 (99.24%)	53.09	43.18	0.4–5.9%	I3
CI = credibility interval						

For each sample, radiocarbon date, the percentage of mtDNA covered at least at 3-fold coverage, average fragment length, deamination at 5′-end, contamination estimate and mitochondrial haplogroup are reported.

**Table 2:** Parameters estimation of the *admixture tot* model.

	Median	Mode	95% HPD-LowB	95% HPD-UppB	R Squared
P	0.872	0.966	0.518	1	0.400
rs	1.647	1	1	4.050	0.088
Nan	2,649	1,517	107	7,626	0.500
Nas	793	461	100	2,785	0.503
Ncn	38,574	14,871	1673	94,089	0.060
Ncs	21,371	9,801	1,000	84,554	0.312
mut	2.1E-08	2E-08	1.3E-08	3.1E-08	0.523

*P* is the proportion of Sardinian lineages coming from Neolithic Europe, *rs* is the extent of population reduction due to the bottleneck of the first colonization of Sardinia, *Nan* is the ancient effective population size of Neolithic Europe, *Nas* is the ancient Sardinian effective population size, *Ncn* is the current European effective population size, *Ncs* is the current Sardinian effective population size and *mut* is the mutation rate per nucleotide per year.
