# Supplementary material for: Complete mitochondrial sequences from Mesolithic Sardinia
Source: Sci Rep. 2017 Mar 3;7:42869. doi: 10.1038/srep42869 (PMC5335606; doi:10.1038/srep42869)
Supplement: Supplementary Material [file srep42869-s1.doc]

**Complete mitochondrial sequences from Mesolithic Sardinia**

Supplementary Material

Alessandra Modi*^1^, Francesca Tassi* ^2^, Roberta Rosa Susca^2^, Stefania Vai^1^, Ermanno Rizzi^3,4^, Gianluca De Bellis^4^, Carlo Lugliè^5^, Gloria Gonzalez Fortes^2^, Martina Lari^1^, Guido Barbujani^2^, David Caramelli*^1^ and Silvia Ghirotto*^2^.

1 Dipartimento di Biologia, Università di Firenze, 50122 Florence, Italy

2 Dipartimento di Scienze della Vita e Biotecnologie, Università di Ferrara, 44121 Ferrara, Italy 3 Fondazione Telethon, 20121 Milano, Italy (current affiliation)

4 Istituto di Tecnologie Biomediche, CNR, 20090 Segrate, Milano, Italy.

5 LASP, Dipartimento di Storia, Beni Culturali e Territorio, Università di Cagliari, 09124 Cagliari, Italy

* These authors contributed equally to this work. Correspondence and requests for materials should be addressed to S.G. (email: ghrslv@unife.it)

**Archaeological Site and samples description**

**Su Carruppu Rockshelter**

The Su Carroppu rockshelter opens onto the Paleozoic massif of the Sulcis region, around 12 km as the crow flies from the present-day South-west coast of Sardinia. Among the currently known Sardinian Early Neolithic (EN) settlements, the Su Carroppu site is one of the southernmost and farthest from the interior. This site has been the first unequivocally recognized as EN at the end of the 1960s, thus becoming for a long time representative of the early stages of the spread of the impressed ware to the Tyrrhenian basin, and eponymous of the corresponding archaeological facies. The 1978 investigation divided the site into two distinct units, called sector A and B. Sector B – covering a surface of only 6 square meters – yielded a stratigraphic sequence representative of the entire site, roughly subdivided into four levels1 (Supplementary Fig. 1). Levels 1 to 3, strongly perturbed by anthropic activities and burrowing animals, confirmed the presence of occupational phases spanning from EN to Bronze Age. The lowermost level, horizon 4, consisted of dark anthropogenic soil, rich in charcoal. This level, between 1.40 and 1.55 mt depth, apparently was preserved undisturbed: basing on the exclusive presence of Cardial Pottery it has been assigned to the EN and yielded large quantities of material remains and bones.

The excavations carried out since 2009 and still in progress are bringing new data to the debate on the chronology and the nature of the ancient human settlement of the site, that had a long term dwelling for at least eight millennia. The revision of older excavations carried out in the Northern sector allowed to identify Mesolithic burials of the IX-VIII millennia cal BCE, suggesting an early settlement before the Neolithic age; furthermore, new analysis of archaeological and anthropological materials from level-4 confirm the presence of a rather ancient Mesolithic frequentation of the island. These new data suggest that Su Carroppu is one of the site which shows the earliest trace of human migrations and frequentations of Sardinia.

**Analyzed samples**

The skeletal remains studied here were brought to light from the base of the undisturbed level-4 during the 1978 excavation season, and were found intermingled and in concretion with bones of Prolagus sardus (the endemic Sardinian pika). Fragments of the ribs as well as from the left and right upper and lower limbs (ulna, radius, humerus, femur, fibula and tibia) have been identified, and some of them were covered by calcareous concretions (Fig. 1). Three direct radiocarbon dates, performed on the human bones of ulna, tibia and humerus labeled CAR-H3, CAR-H7 and CAR-H8 respectively, placed the remains in the mid-9^th^ millennium cal. BCE thus showing an unexpected Early Mesolithic settlement predating EN occupation^2,3^(Supplementary Table S1).

**Methods Ancient DNA**

The ancient DNA (aDNA) work was carried out at the exclusively dedicated aDNA facilities at University of Florence, Laboratory of Anthropology, and following appropriate criteria to prevent/minimize contamination with present-day DNA. DNA extraction and library preparation reactions included negative controls.

Samples preparation.

Molecular analysis was performed on the dated fragments CAR-H3, CAR-H7 and CAR-H8. All sample-preparation steps have been executed following strictly specific ancient DNA requirements. Before starting with sample processing, calcareous concretions that covered the bones were removed using sterile scalpels and abrasive disks, the outer layer of bones was removed with a dental drill and then the bones were irradiated for 1 hour under UV lights (260 nm). Samples were ground to fine powder using a dental drill rotated at low speed and usually stored at -20°C.

DNA extraction and library preparation.

For each sample, DNA was extracted from 100 mg of bone powder following a published silica-based protocol especially designed for the retrieve of molecules as short as 50 base pair (bp)^4^. DNA was eluted in 100 µl of TE buffer (10 nM Tris, 1 mM EDTA) with 0.05% Tween-20 (TET buffer), and stored at -20°C until use.

Twenty-five µl of each extract were converted into double-strand Illumina library without enzymatic damage repair in order to preserve the damage patterns of DNA fragments^5^. A unique combination of two indexes was added to both ends of the each library molecules as sample specific DNA barcodes by 10 cycles of PCR. The entire library volume was split in 4 reactions and was used as templates in 100 µl PCR reactions containing 10x Pfu reaction Buffer, 2.5 U PfuTurbo DNA polymerase (Agilent Technologies), 250 µM each dNTP and 200 nM each indexing primers. Cycling conditions were comprised of an activation step lasting 2 min at 95°C, followed by 10 cycles of denaturation at 95°C for 30 sec, annealing at 58°C for 30 sec and elongation at 72°C for 1 min, with a final extension step at 72°C for 10 min. PCR products were purified using the MinElute PCR Purification Kit (QIAGEN) and eluted in 50 µl TET buffer. An aliquot of each library before and after indexing PCR was quantified using qPCR and the indexing reaction success was determined in term of number of indexed molecules over the total number of molecules without indexes (Supplementary Table S11). Extraction and library negative controls were treated accordingly and show concentrations of at least 4 orders of magnitude lower than samples in all quantified steps (Supplementary Table S11). Libraries were amplified to reach plateau with different number of PCR cycles in order to reduce the heteroduplex complexes, using AccuPrime Pfx DNA polymerase as described in Schuenemann et al. (2013)^6^. Amplified products were purified with MinElute PCR Purification Kit (QIAGEN) and eluted in 20 ul TET buffer and were then analyzed on Agilent 2100 Bioanalyzer (DNA 1000 chip) in order to estimate the quality and the quantity of the produced libraries.

Mitochondrial DNA capture and sequencing.

The libraries were enriched for human mitochondrial DNA (mtDNA) in a bead-capture method using long-range PCR products as probes for hybridization as described in Maricic et al (2010)^7^. Two µg of each indexed library were mixed with 500 ng of biotinylated DNA baits and were incubated at 65°C. Indexed libraries from extraction and library negative controls were pooled equimolarly and captured together. After 48 hours, molecules that did not hybridize to the bait were washed away while the enriched libraries were eluated with 10mM NaOH and purified on MinElute spin columns (QIAGEN) with final elution in 15 µl of TET buffer. The DNA concentration was measured by qPCR (Supplementary Table S12) and the captured libraries were further amplified to reach plateau using AccuPrime Pfx DNA polymerase as above. Enriched libraries were quantified using an Agilent 2100 Bioanalyzer DNA 1000 chip, pooled in equal concentration and sequenced on Illumina MiSeq. High-throughput DNA sequencing was carried out at the Institute of Biomedical Technologies, National Research Council, in Segrate (Milano). The sequencing was run as paired- end with 2 × 75 + 8 + 8 cycles. Base calls were done on instrument during the sequencing run by Real Time Analysis (RTA) software. The generated .bcl files were converted in compressed FASTQ files and demultiplexed using CASAVA-1.8.2. In total we generated 1,639,184 reads for the samples CAR-H3, CAR-H7 and CAR-H8 (Supplementary Table S12).

Raw reads processing, mapping, consensus call.

Raw reads were processed with SeqPrep^8^. Adaptor sequences were trimmed and paired-end reads were merged into single sequences with a minimum overlap of 10 bp in order to exclude all the sequences derived from molecules longer than 140 bp. Only reads with a minimum length of 30 bp were kept. Filtered reads were mapped to the revised Cambridge Reference Sequence (rCRS, NC_012920.1) using BWA-0.6.2^9^. To improve the mapping efficiency in ancient molecules, we deactivated the seeding and we allowed more substitutions and up to two gaps (instead of 1) setting “-l 1000 -n 0.01 -o 2”^10^. PCR duplicates were removed using PicardTools-1.98 (http://picard.sourceforge.net.). The mapped reads were filtered based on mapping quality ≥ 30 using SAMtools-0.1.19^11^ and to align to unique positions along the reference sequence. RealignerTargetCreator and IndelRealigner from the suite of tools Genome Analysis Tool Kit (GATK v. 3.0)^12^ were used to identify regions that contain an implausibly large number of differences to the reference genome and then to realign sequences in these regions.

Consensus sequences for the mitochondrial genomes of all samples were called using mpileup and vcfutils.pl of the SAMtools-0.1.19 package^9^. Only the reads with a minimum mapping quality of 30 were used to call confident bases for these consensus sequences. Finally, we obtained an average coverage of mtDNA of 4.83 for CAR-H3, 14.14 for CAR-H7 and 19.98 for CAR-H8 with average fragment length of 72.74 for CAR-H3, 62.59 for CAR-H7 and 53.09 for CAR-H8 (Table 1 and Supplementary Table S13). Due to the low coverage obtained, sample CAR-H3 was excluded to further analysis. A summary of each library can be found in Supplementary Table S12.

To avoid miscalling, all the polymorphic positions (Supplementary Table S4) reported in the vcf output file

were subsequently visually inspected. The assembly to the reference was visualized in Tablet^13^ and we

masked all the positions covered by less than three reads. We applied the IUPAC code, when the

concordance across reads was lower than 70%. The haplotype assignment was based on Haplogrep^14, 15^.

Contamination estimates.

The authenticity of the consensus sequences was evaluated by the analysis of DNA damage patterns and by identifying the specific diagnostic positions that differ from a set of 311 modern human mtDNAs. It has

been shown that over time cytosine will deaminate to uracil, which code as thymine residues16, especially at the ends of the fragments. The C to T misincorporation patterns at the 5’ end of the molecules can be exploited to test for authenticity of aDNA sequences17,18. To do this, we used mapDamage2.019 and, as expected for double strand library, we observed an increase of C to T on the 5’ end and G to A on the 3’ end. Both samples showed a substitution rate higher than 30%, and therefore fully compatible with ancient DNA degradation (Supplementary Fig. S2 and Supplementary Table S2).

To estimate contaminant/authentic mixture proportion in mitochondrial sequence data, we used the likelihood-based method described in Fu et al. (2013)^20^. We computed the Bayesian contamination estimate comparing the reads of each ancient sample with the respective consensus sequences and 311 present-day human mitochondrial genomes, which represent all possible contaminating sequences. The proportion of authentic reads was high in both samples, i.e. 95% for CAR-H7 and 98% for CAR-H8 (Supplementary Fig. S3 and Supplementary Table S2).

**Dataset of ancient and modern sequences**

To study the two Mesolithic Sardinian samples in the context of the ancient genetic diversity, we analyzed 49 already published pre-Neolithic sequences, coming from all over Europe. We classified this ancient dataset in four chronologically-based groups: pre-LGM (ranging from 45 to 25 kya), post-LGM (ranging from 19.5 to 14.5 kya), Late Glacial (ranging from 14.5 to 11.5 kya) and Holocene (ranging from 11.5 to 7 kya). The sequences we used are listed in Supplementary Table S5.

Then, we created a dataset to analyse the genetic variation within the haplogroup J2b. We examined an enlarged whole-genome mitochondrial database, and we collected 48 modern and 5 ancient samples belonging to this haplogroup. The details of each sample are reported in Supplementary Table S6.

Finally, to shed light on the Sardinian past demographic history through the Approximate Bayesian Computation framework, we combined our two Mesolithic Sardinians samples with 46 high quality Neolithic mtDNA sequences from Germany (28 Early Neolithic and 18 Middle Neolithic^21^) that represented, in our models, a source of European Neolithic genetic variation, and with 63 modern sample from Ogliastra^22^ (a Sardinian region). The Ogliastra sample is, at the best of our knowledge, the unique unbiased sample of Sardinian complete mitochondrial genomes available. Unfortunately, although many mitochondrial complete genomes are now publicly available, they suffer from the fact that the samples have been first screened by sequencing hypervariable segments of the mtDNA control region to classify haplogroups and then only specific haplogroups have been selected for complete mtDNA genome sequencing. Unfortunately, such sampling is biased, not representative of the population, and thus not suitable for demographic inference. The Ogliastra sample we used, besides being unbiased, has already shown genetic continuity with an island-wide Bronze Age sample, and thus can be considered too well represent the Sardinian genetic background. A summary of the dataset is shown in Supplementary Table S14. In order to minimize the bias, when the row reads were available, we applied to each ancient sample the same processing pipeline used for the analysis of the two Mesolithic Sardinians. The software MUSCLE^23^ was used to align the sequences included in each dataset. We excluded from the analysis the A/C stretch length polymorphism in regions 16180–16193 and 303–315.

**Approximate Bayesian Computation**

We simulated three demographic models (Fig. 3), all comprising two populations that separated 20,000 years ago. After this separation there are two branches, that for sake of simplicity we shall call “Sardinia” and “continental Europe”. In all the three models, the “continental Europe” branch remains of constant size until 12,000 years ago, then the growth is exponential until present time. The Sardinia branch underwent a bottleneck around 19,000 years ago (representing the first settling of the island^24^), bottleneck that lasts until 6,000 years ago. In the *continuity* model, the two branches remain separated all the time, and the Neolithization of the Sardinian branch does not involve genetic exchanges with continental Europe. Five- thousand years ago starts an exponential growth that lasts until present time and the continuity within the island is complete since the first settlement. In the *discontinuity* model there is a complete replacement of ancient Sardinians by Neolithic lineages coming from continental Europe 8,000 years ago. To this time, the Mesolithic Sardinian population starts to decline, whereas the newly migrated, Neolithic, population starts to growth until present time. In the *admixture* model, the current inhabitants of Sardinia are a mixture of local (i.e. Mesolithic) and Neolithic lineages, the latter coming mostly 6,000 years ago concurrently with the demographic increase. The proportion of Neolithic lineages giving rise to modern Sardinians is regulated by the parameter *P*, which in this model is free to vary from 0.1 and 0.8. The *admixture_tot* model is defined in the same way of the *admixture* one, but for the parameter P, which that in this case has an associated prior distribution ranging from 0 to 1. The parameters that are shared between the models have the same prior distributions; the complete list of models' parameters and of the associated prior distributions are presented in Supplementary Table S13. For each model we ran 500,000 simulations.

We summarized the data by means of summary statistics (the number of haplotypes, the haplotype diversity, the total and the private number of segregating sites, the Tajima's D, and the average number of pairwise differences for each population; the Fst and the mean number of differences between pairs of populations to summarize the variation between populations), calculated with *arlsumstat*^25^. When calculating models' posterior probabilities, we plotted for each summary statistic the distribution of values generated by each model, and we evaluated, by visual inspection, which statistics were able to better distinguish between models. We then used this subset of statistics (the total and private number of segregating sites, the Tajima's D within samples and the Fst between pairs of sample) to calculate the presented posterior probabilities. When estimating model's parameters we reduced the dimensionality of the space of statistics via Partial Least Square ( PLS ) transformation^26^. Under this approach, we defined a set of orthogonal linear- combinations of summary statistics best explaining the variance in the model parameter space. After the inspection of the Root Mean Square Error Plots, we selected 10 PLS to calculate the posterior probabilities of model's parameters.

**SUPPLEMENTARY FIGURES**

**Supplementary Figure S1**. The sampling location of analyzed remains. (a) Su Carroppu site (b) archaeological site during the excavation season (c) stratigraphic profile in the sector B of the excavation; samples analyzed in this study were retrieved in the lowermost level, horizon 4 (in red).


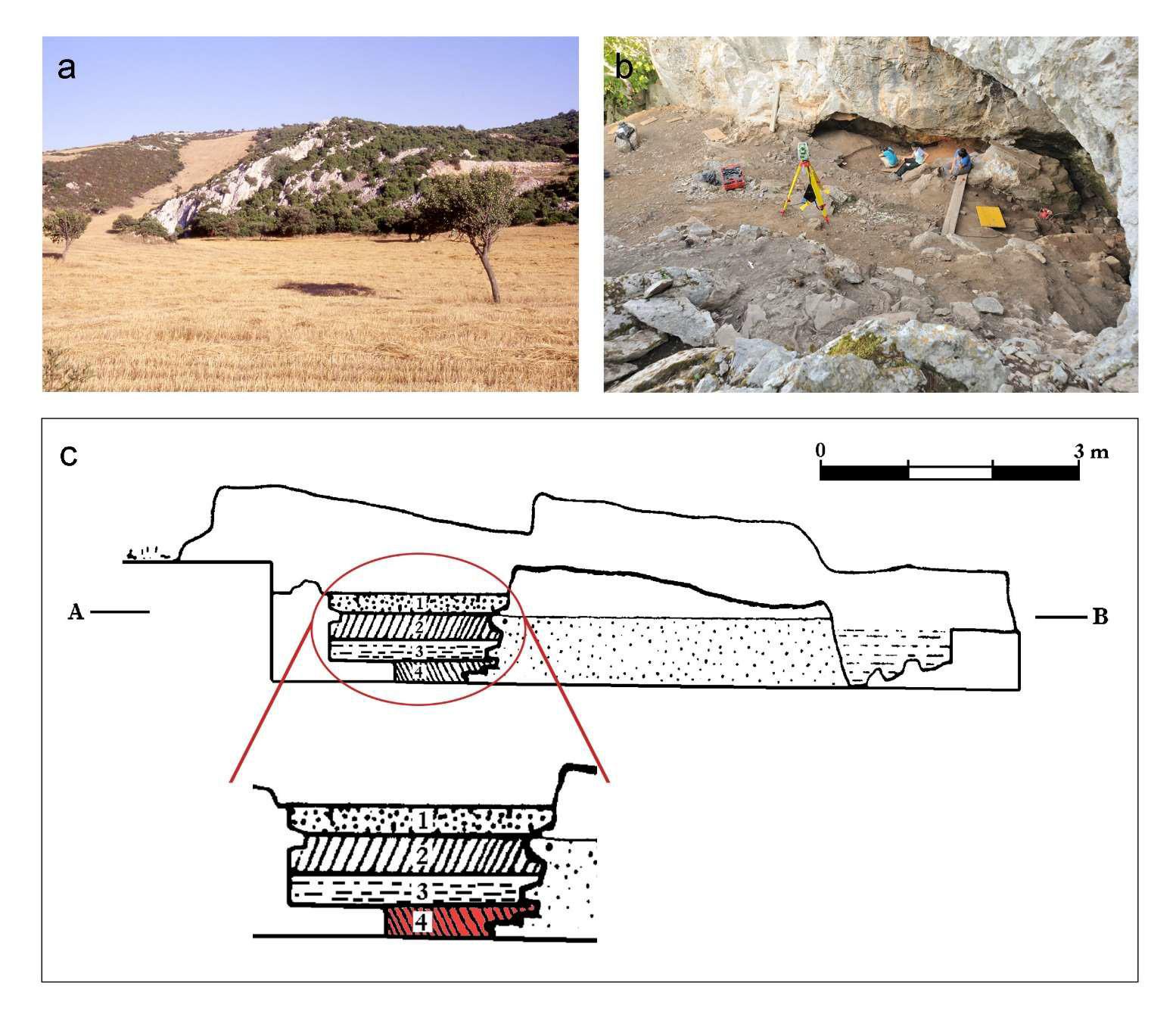


**Supplementary Figure S2.** Damage patterns of analyzed samples. (a) for CAR-H7 (b) for CAR-H8. Mismatch frequency to the reference as function of nucleotide position within the read. C→T in red and G→A in blue.


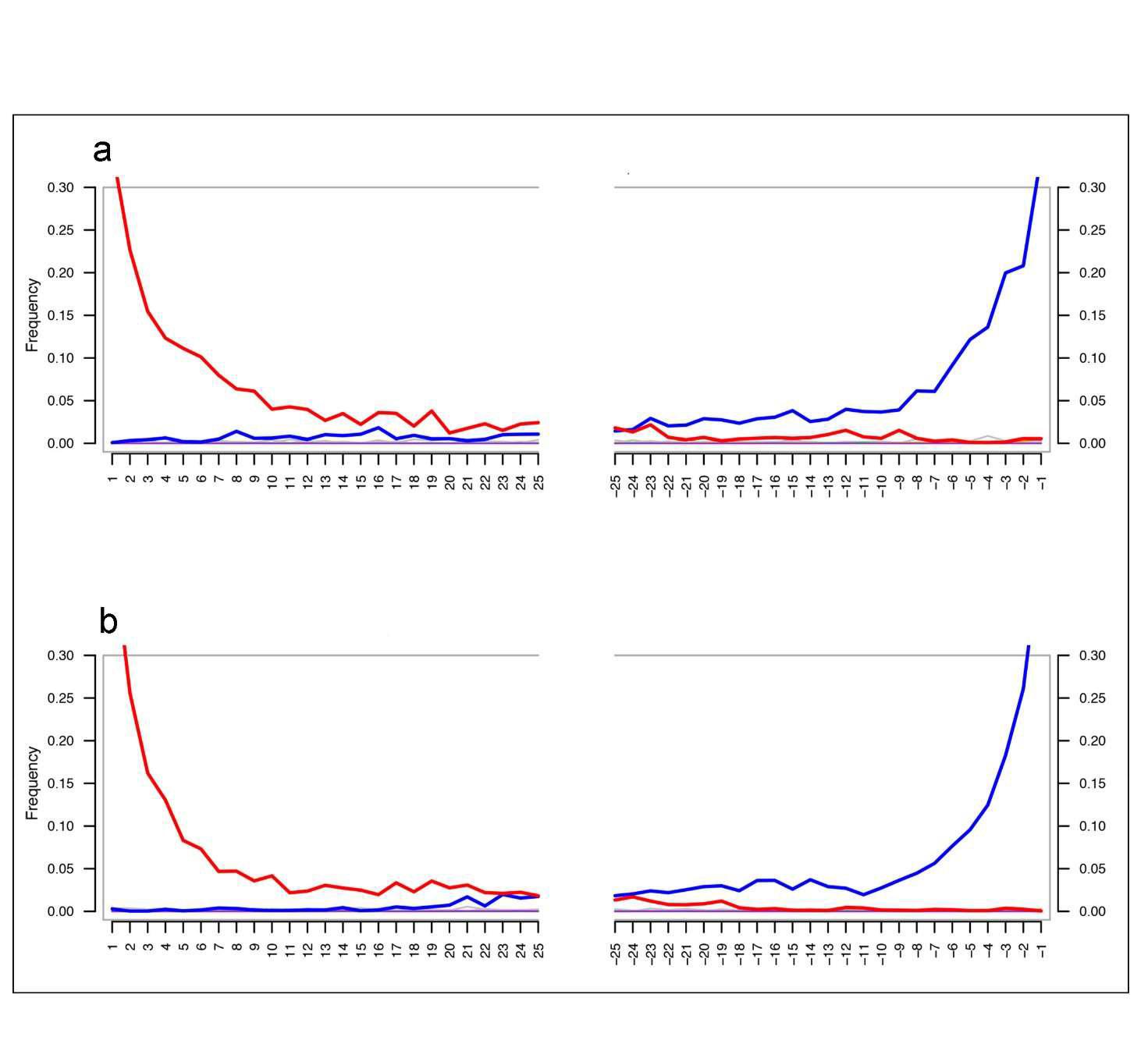


**Supplementary Figure S3.** Results of Likelihood-based contamination estimates. (a) for CAR-H7. (b) for CAR-H8. Estimated posterior density for the proportion of authentic reads.


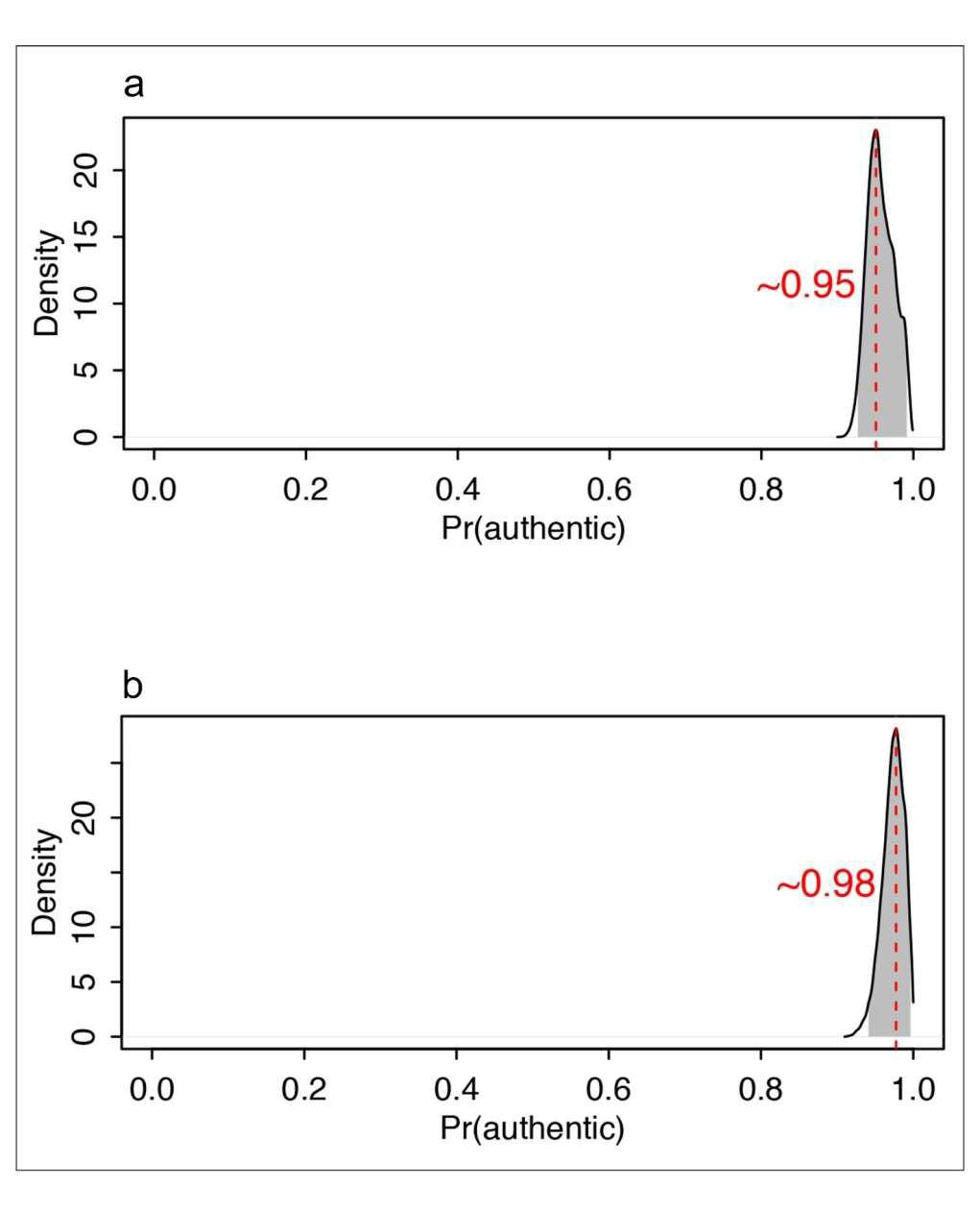


**Supplementary Figure S4**. Parameters’ Posterior Distributions of the *admixture* model. The X axis covers the range of the (uniform) prior distributions. *P* is the proportion of Sardinian lineages coming from Neolithic Europe, *rs* is the extent of population reduction due to the bottleneck of the first colonization of Sardinia, *Nan* is the ancient effective population size of Neolithic Europe, *Nas* is the ancient Sardinian effective population size, *Ncn* is the current European effective population size, *Ncs* is the current Sardinian effective population size and *mut* is the mutation rate per nucleotide per generation.


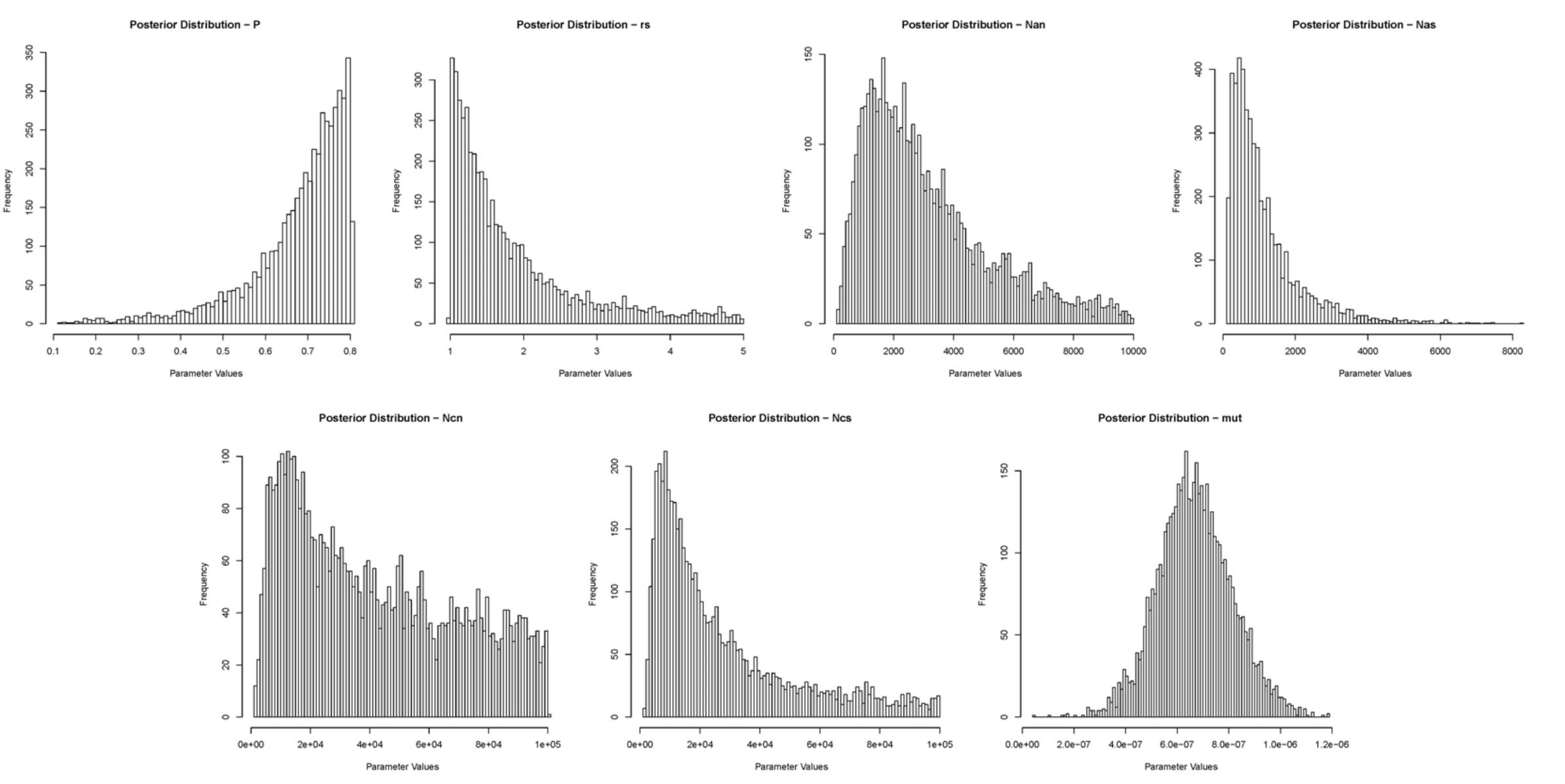


**Supplementary Figure S5**. Principal Component Analysis of the 5,000 best simulations under each of the three models compared via ABC.


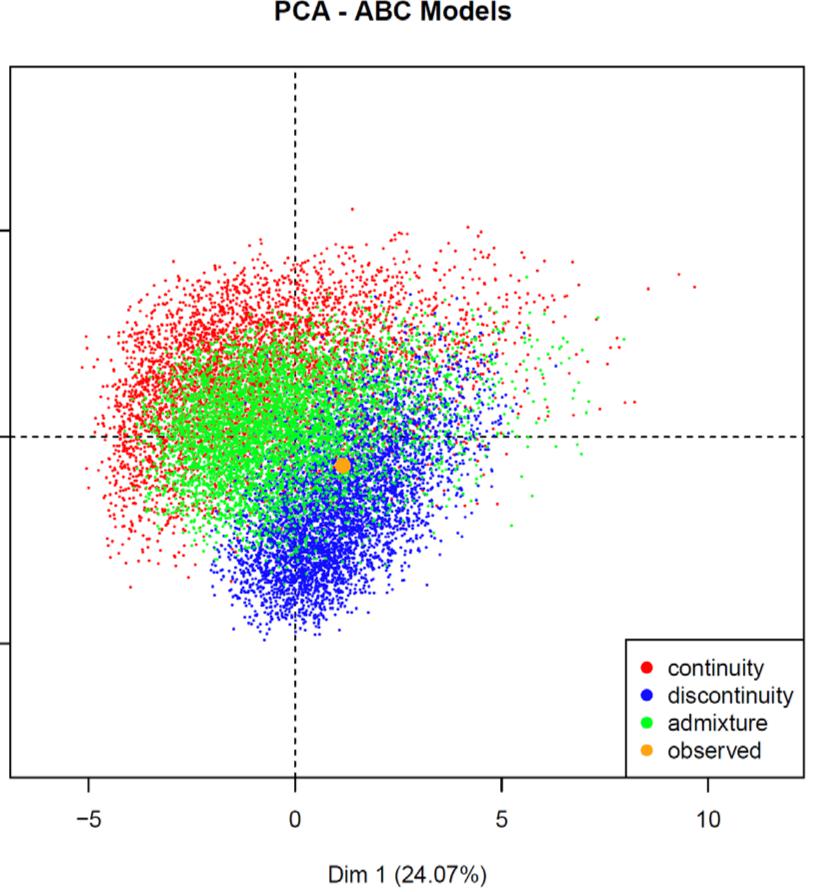


**Supplementary Figure S6.** Parameters’ Posterior Distributions of the *admixture _tot* model. The X axis covers the range of the (uniform) prior distributions. *P* is the proportion of Sardinian lineages coming from Neolithic Europe, *rs* is the extent of population reduction due to the bottleneck of the first colonization of Sardinia, *Nan* is the ancient effective population size of Neolithic Europe, *Nas* is the ancient Sardinian effective population size, *Ncn* is the current European effective population size, *Ncs* is the current Sardinian effective population size and *mut* is the mutation rate per nucleotide per generation.


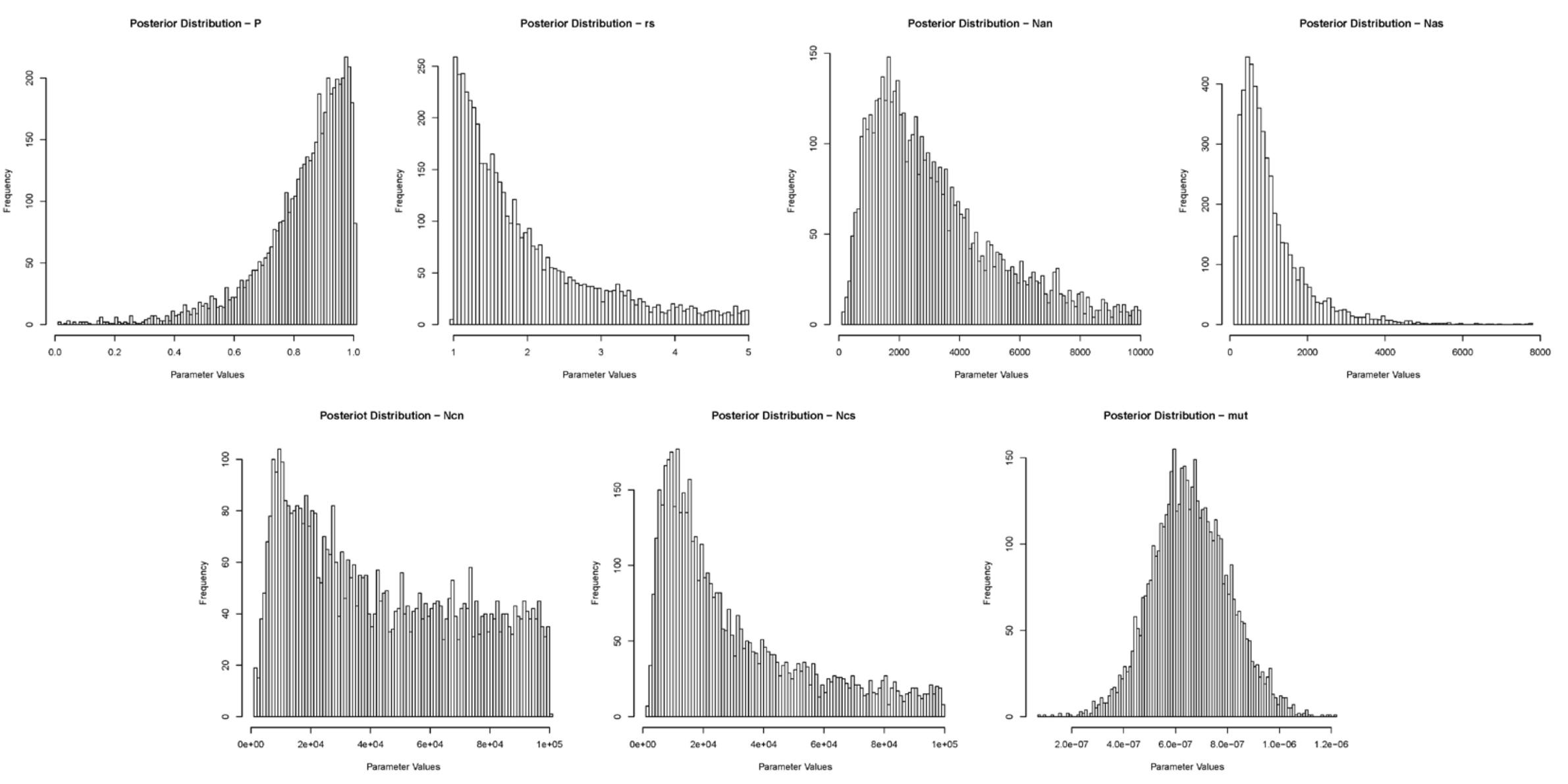


**SUPPLEMENTARY TABLES**

Supplementary Table S1 – Radiocarbon dates of samples.

|  |  |  |  |  |  | 2σ cal. age |
| --- | --- | --- | --- | --- | --- | --- |
| Sample ID | Service reference | Context | Matter | ^14^C age (BCE) | St. dev. | (BCE) |
| CAR-H3 | AA-75645 | testB-lev 4, bottom | Ulna | 8620 | 80 | 7938-7525 |
| CAR-H7 | AA-80544 | testB-lev 4, bottom | Tibia | 8780 | 130 | 8227-7596 |
| CAR-H8 | AA-80545 | testB-lev 4, bottom | Humerus | 9200 | 180 | 9124-7851 |

**Supplementary Table S1.** Radiocarbon dates of samples. The table gives the archaeological context, anatomical element, uncalibrated and calibrated 14C-dates for all the analyzed samples. The service reference AA is from NSF Arizona AMS Facility, University of Arizona, Tucson AZ. Calibrated dates are computed from the OxCal computer program (v4.2).

Supplementary Table S2 – Contamination estimates.

|  |  |  |  | Contamination estimate results | | | | |
| --- | --- | --- | --- | --- | --- | --- | --- | --- |
|  |  | mapDamage results | | (Bayesian Estimate) | | | | |
|  |  |  |  | Contamination | proportion | |  | |
|  |  | C->T misincorporation | G->A misincorporation | estimate | (authentic) | | | |
|  | Sample ID | at 5’-end (%) | at 3’-end (%) | (95% CI) |  |  | |  |
|  |  |  |  |  |  |  | |  |
|  | CAR-H3 | N/A | N/A | N/A | N/A |  | | |
|  | CAR-H7 | 34.45 | 33.66 | 0.9% - 7.3% | 0.950 |  | |  |
|  | CAR-H8 | 43.18 | 42.80 | 0.4% - 5.9% | 0.977 |  | |  |

CI = credibility interval

**Supplementary Table S2**. Contamination estimates (related to Supplementary Fig. S2 and Supplementary Fig. S3). mtDNA contamination estimates based on misincorporation patterns at the ends of the fragments using mapDamage2.0^19^ and on Bayesian framework (contamMix)^20^. (See “Contamination

estimate” in Supplementary Methods – Ancient DNA).

Supplementary Table S3 – Estimation of contamination rate in haplogroup-defining positions

|  | Sample | Total | Total | Secondary | Potentially | %C+MD | %C |
| --- | --- | --- | --- | --- | --- | --- | --- |
|  |  | reads | variants | variants | damaged bases |  |  |
|  |  |  |  |  |  |  |  |
|  | CAR-H7 | 423 | 35 | 23 | 6 | 5.44 | 4.02 |
|  | CAR-H8 | 414 | 27 | 26 | 12 | 6.28 | 3.38 |

**Supplementary Table S3.** Estimation of contamination rate in haplogroup-defining positions, %C+MD: percentage of contamination including sites with potentially damaged bases; %C: percentage of contamination excluding sites with potentially damaged bases.

Supplementary Table S4 – mtDNA variants respect to the rCRS.

|  | Sample ID | Range | Haplogroup |  |  |  | Polymorphisms | | | |  |  |  |
| --- | --- | --- | --- | --- | --- | --- | --- | --- | --- | --- | --- | --- | --- |
|  | CAR-H7 | 1-16569 | J2b1 | 73G 150T 152C **195C** 263G 295T 489C 750G 1438G | | | | | | | | | |
|  |  |  |  | 2706G | **3654T** | 4216C | | 4769G | | 5633T | | **6053T** | **6959T** |
|  |  |  |  | 7028T 7476T 8860G **8953G 9071T** 10172A 10398G | | | | | | | | | |
|  |  |  |  | **10957G** | 11251G | | 11719A | | 12612G | | 13708A | | 14766T |
|  |  |  |  | 15257A 15326G 15452A 15812A 16069T 16126C | | | | | | | | | |
|  |  |  |  |  |  |  |  |  |  |  |  |  |  |
|  | CAR-H8 | 1-16569 | I3 | 73G 152C 239C | | | 250C 263G | | | 750G | | 1438G | 1719A |
|  |  |  |  | 2706G 4529T 4769G 7028T 8251A 8860G 10034C | | | | | | | | | |
|  |  |  |  | 10238C | 10398G | | 11719A | | 12501A | | 12705T | | 14766T |
|  |  |  |  | 15043A 15326G 15924G 16223T 16391A 16519C | | | | | | | | | |

**Supplementary Table S4.** MtDNA variants respect to the rCRS. Private polymorphisms are highlighted in bold.

Supplementary Table S5 – Dataset of the Pre-Neolithic samples.

|  | Sample | ID | Haplogroup | Time period | Country | Reference |
| --- | --- | --- | --- | --- | --- | --- |
|  | BERRYAUBAC1 | BEAUBAC1 | U5b1a | Holocene | France | 27 |
|  | BICHON | BICHON | U5b1h | LateGlacial | Switzerland | 28 |
|  | BLA 20 | BLA20 | U5a2c3 | Holocene | Germany | 29 |
|  | BOCKSTEIN | BOCKST | U5b1d1 | Holocene | Germany | 27 |
|  | BRILLENHOHLE | BRILLEN | U8a | post-LGM | Germany | 27 |
|  | BURKHARDTSHO | BURKLE | U8a | post-LGM | Germany | 27 |
|  | CIOCLOVINA1 | CIOCLO1 | U | pre-LGM | Romania | 27 |
|  | CUIRYLESCHAUD | CRYCHAU1 | U5b1b | Holocene | France | 27 |
|  | DOLNIVESTONICE | DOVE13 | U8 | pre-LGM | Czech Republic | 20 |
|  | DOLNIVESTONICE | DOVE14 | U | pre-LGM | Czech Republic | 20 |
|  | DOLNIVESTONICE | DOVE16 | U5 | pre-LGM | Czech Republic | 27 |
|  | DOLNIVESTONICE | DOVE43 | U5 | pre-LGM | Czech Republic | 27 |
|  | FALKENSTEIN | FALKNST | U5b2a | Holocene | Germany | 27 |
|  | FELSDACH | FELSDACH | U5a2c | Holocene | Germany | 27 |
|  | FUMANE2 | FUMA2 | R | pre-LGM | Italy | 30 |
|  | GOYET2878-21 | GY2878 | U5 | pre-LGM | Belgium | 27 |
|  | GOYETQ116-1 | GYQ116-1 | M | pre-LGM | Belgium | 27 |
|  | GOYETQ-2 | GYQ-2 | U8a | post-LGM | Belgium | 27 |
|  | GOYETQ376-19 | GYQ376-19 | U2 | pre-LGM | Belgium | 27 |
|  | GOYETQ376-3 | GYQ376-3 | M | pre-LGM | Belgium | 27 |
|  | GOYETQ53-1 | GYQ53-1 | U2 | pre-LGM | Belgium | 27 |
|  | GOYETQ55-2 | GYQ55-2 | U2 | pre-LGM | Belgium | 27 |
|  | GOYETQ56-16 | GYQ56-16 | U2 | pre-LGM | Belgium | 27 |
|  | HOHLEFELS10 | HOFE10 | U8a | post-LGM | Germany | 27 |
|  | HOHLEFELS49 | HOFE49 | U8a | post-LGM | Germany | 27 |
|  | HOHLEFELS79 | HOFE79 | U8a | post-LGM | Germany | 27 |
|  | HOHLENSTEINSTA | HOHLSTAD | U5b2c1 | Holocene | Germany | 27 |
|  | IBOUSSIERES25-1 | IBO25-1 | U5b2a | LateGlacial | France | 27 |
|  | IBOUSSIERES31-2 | IBO31-2 | U5b1 | LateGlacial | France | 27 |
|  | IBOUSSIERES39 | IBO39 | U5b2b | LateGlacial | France | 27 |
|  | KOSTENKI14 | KOST14 | U2 | pre-LGM | Russia | 31 |
|  | KOTIAS | KOTIAS | H13c | Holocene | Georgia | 28 |
|  | LABRANA | LABRANA | U5b2c1 | Holocene | Spain | 32 |
|  | LAROCHETTE | LAROCHE | M | pre-LGM | France | 27 |
|  | LESCLOSEAUX3 | LECLX3 | U5a2 | Holocene | France | 27 |
|  | LOSCHBOUR | LOSCHB | U5b1a | Holocene | Luxembourg | 33 |
|  | MA1 | MA1 | U | pre-LGM | Russia | 34 |
|  | MAREUILLESMEA | MARMX1 | U5a2 | Holocene | France | 27 |
|  | OASE1 | OASE1 | N | pre-LGM | Romania | 30 |
|  | OBERKASSEL998 | OBER998 | U5b1 | LateGlacial | Germany | 20 |
|  | OFNET | OFNET | U5b1d1 | Holocene | Germany | 27 |
|  | PAGLICCI108 | PAG108 | U2'3'4'7'8'9 | pre-LGM | Italy | 27 |
|  | PAGLICCI133 | PAG133 | U8c | pre-LGM | Italy | 27 |
|  | PAGLICCI71 | PAG71 | U5b2b | post-LGM | Italy | 27 |
|  | RANCHOT88 | RANCH88 | U5b1 | Holocene | France | 27 |
|  | RIGNEY1 | RIGNEY1 | U2'3'4'7'8'9 | post-LGM | France | 27 |
|  | ROCHEDANE | ROCHE | U5b2b | LateGlacial | France | 27 |
|  | SATSURBLIA | SATSBL | K3 | LateGlacial | Georgia | 28 |
|  | USTISHIM | UST | R | pre-LGM | Russia | 35 |
|  |  |  |  |  |  |  |

**Supplementary Table S5**. Dataset of Pre-Neolithic samples.

Supplementary Table S6 – Dataset of the ancient and modern samples belonged to the haplogroup J2b.

|  | Sample | Time period | Country | Reference |
| --- | --- | --- | --- | --- |
|  | ALB_2 | Modern | Albania | 36 |
|  | ARM_10 | Modern | Armenia | 37 |
|  | CRE_8 | Modern | Crete | 36 |
|  | CRE_9 | Modern | Crete | 36 |
|  | DEN_1 | Modern | Denmark | 38 |
|  | ENG_3 | Modern | England | 39 |
|  | ENG_6 | Modern | England | 39 |
|  | FIN_1 | Modern | Finland | 38 |
|  | FIN_2 | Modern | Finland | 40 |
|  | FIN_3 | Modern | Finland | 40 |
|  | GRE_17 | Modern | Greece | 36 |
|  | GRE_18 | Modern | Greece | 36 |
|  | IRAN_11 | Modern | Iran | 36 |
|  | IRE_1 | Modern | Ireland | 41 |
|  | IRE_3 | Modern | Ireland | 41 |
|  | IRE_6 | Modern | Ireland | 39 |
|  | ITA_1 | Modern | Italy | 38 |
|  | ITA_6 | Modern | Italy | 41 |
|  | ITA_46 | Modern | Italy | 36 |
|  | ITA_47 | Modern | Italy | 36 |
|  | ITA_48 | Modern | Italy | 36 |
|  | ITA_49 | Modern | Italy | 36 |
|  | ITA_50 | Modern | Italy | 36 |
|  | ITA_51 | Modern | Italy | 36 |
|  | ITA_52 | Modern | Italy | 36 |
|  | ITA_53 | Modern | Italy | 36 |
|  | ITA_54 | Modern | Italy | 36 |
|  | POR_4 | Modern | Portugal | 39 |
|  | ROM_9 | Modern | Romania | 36 |
|  | RUS_10 | Modern | Russia | 36 |
|  | RUS_13 | Modern | Russia | 36 |
|  | RUS_7 | Modern | Russia | 36 |
|  | RUS_9 | Modern | Russia | 36 |
|  | SAR_2 | Modern | Sardinia (Italy) | 42 |
|  | SAR_4 | Modern | Sardinia (Italy) | 42 |
|  | SAR_5 | Modern | Sardinia (Italy) | 43 |
|  | SAR_20 | Modern | Sardinia (Italy) | 36 |
|  | SAR_21 | Modern | Sardinia (Italy) | 36 |
|  | SAR_22 | Modern | Sardinia (Italy) | 36 |
|  | SAR_23 | Modern | Sardinia (Italy) | 36 |
|  | SIB_1 | Modern | Siberia | 44 |
|  | SLO_4 | Modern | Slovakia | 36 |
|  | SPA_12 | Modern | Spain | 45 |
|  | SPA_13 | Modern | Spain | 45 |
|  | SPA_14 | Modern | Spain | 45 |
|  | SYR_1 | Modern | Syria | 36 |
|  | SYR_2 | Modern | Syria | 36 |
|  | SYR_3 | Modern | Syria | 36 |
|  | Rathlin3(RSK2) | Bronze age | Ireland | 46 |
|  | RISE524 | Bronze age | Russia | 47 |
|  | RISE392 | Bronze age | Russia | 48 |
|  | I0423 | Bronze age | Russia | 48 |
|  | I1302 | Neolithic | Spain | 48 |

**Supplementary Table S6.** Dataset of the ancient and modern samples belonged to the haplogroup J2b.

Supplementary Table S7 - ABC models’ Posterior Probabilities calculated considering different numbers of retained.

|  | Threshold | Continuity | Discontinuity | Admixture |
| --- | --- | --- | --- | --- |
|  | 50,000 | 0.001 | 0.777 | 0.222 |
|  | 100,000 | 0.002 | 0.781 | 0.217 |
|  | 150,000 | 0.002 | 0.785 | 0.213 |
|  |  |  |  |  |

**Supplementary Table S7.** ABC models’ Posterior Probabilities calculated considering different numbers of retained simulations (“Threshold”).

Supplementary Table S8 - Parameters estimation of the admixture model.

|  |  | Median | Mode | 95% HPD-LowB | 95% HPD-UppB | R Squared |
| --- | --- | --- | --- | --- | --- | --- |
|  | P | 0.714 | 0.775 | 0.43 | 0.82 | 0.269 |
|  | rs | 1.54 | 1 | 1 | 3.93 | 0.093 |
|  | Nan | 2,573 | 1,447 | 100 | 7,571 | 0.641 |
|  | Nas | 819 | 415 | 100 | 3,157 | 0.660 |
|  | Ncn | 36,167 | 14,297 | 1,000 | 92,158 | 0.193 |
|  | Ncs | 18,652 | 8,623 | 1,000 | 81,415 | 0.395 |
|  | mut | 2.2E-08 | 2.1E-08 | 1.3E-08 | 3.1E-08 | 0.640 |
|  |  |  |  |  |  |  |

**Supplementary Table S8.** Parameters estimation of the admixture model. *P* is the proportion of Sardinian lineages coming from Neolithic Europe, *rs* is the extent of population reduction due to the bottleneck of the first colonization of Sardinia, *Nan* is the ancient effective population size of Neolithic Europe, *Nas* is the ancient Sardinian effective population size, *Ncn* is the current European effective population size, Ncs is the current Sardinian effective population size and *mut* is the mutation rate per nucleotide per year.

Supplementary Table S9 - ABC power analyses of the continuity, discontinuity and admixture models.

|  | True model | TP | FP |
| --- | --- | --- | --- |
|  | Continuity | 0.792 | 0.084 |
|  | Discontinuity | 0.886 | 0.038 |
|  | Admixture | 0.641 | 0.157 |
|  |  |  |  |

**Supplementary Table S9.** ABC power analyses of the continuity, discontinuity and admixture models. The table reports the true positive (TP) and false positive (FP) rates when comparing the three models using each time 1,000 PODs.

Supplementary Table S10 – Libraries concentration

| Sample ID | Not indexed library^1^ | Indexed library^1^ | Efficiency^2^ | Captured library^1^ |
| --- | --- | --- | --- | --- |
| CAR-H3 | ~5.93E+9 | ~1.60E+12 | 269 | ~1.22E+7 |
| CAR-H7 | ~7.06E+9 | ~2.48E+12 | 350 | ~1.24E+7 |
| CAR-H8 | ~5.09E+9 | ~1.97E+12 | 387 | ~5.01E+6 |
| LB | ~4.29E+5 | ~1.30E+8 | 300 | ~1.14E+5 |
| EB | ~5.27E+5 | ~5.93E+8 | 309 |
|  |

1 = concentration is expressed in copies/µl

2 = Efficiency -> Indexed library/Not indexed library LB = library blank

EB = extraction blank

**Supplementary Table S10**. Libraries concentration. Libraries concentrations before and after indexing reaction. Efficiency of indexing PCR is determined in term of the number of indexed molecules over the total number of molecules without indexes. The table gives also the concentration of the libraries after the mtDNA capture (indexed libraries from extraction and library negative controls were captured together).

Supplementary Table S11 – Sequencing and mapping summary

|  |  |  |  |  | nt covered at |  |
| --- | --- | --- | --- | --- | --- | --- |
|  | Raw reads | Merged reads | Mapped (Q30) | Duplicates | 3-fold coverage | Average |
| Sample ID | (R1 and R2) | (% of merged) | longer than 30 bp | removed | (% of mtDNA) | coverage |
|  |  |  |  |  |  |  |
| CAR-H3 | 682,482 | 328,756 (92.95%) | 3,993 | 1,120 | 13,730 (82.88%) | 4.826 |
| CAR-H7 | 732,682 | 353,396 (93.18%) | 11,741 | 3,846 | 16,527 (99.75%) | 14.123 |
| CAR-H8 | 224,020 | 107,054 (91,53%) | 16,662 | 6,274 | 16,446 (99.24%) | 19.985 |
| Total | 1,639,184 | 789,206 | 32,396 | 11,240 |  |  |

**Supplementary Table S11.** Sequencing and mapping summary. Sequencing and bioinformatics analyses output comprising number of row reads, number of merged reads, number of mapping reads before and after removing PCR duplicates, mtDNA covered at least at 3-fold coverage and average coverage.

Supplementary Table S12 - Prior distribution associated to models’ parameters

|  | shape | Lower boundary | Upper boundary |
| --- | --- | --- | --- |
| P* | uniform | 0.1 (0) | 0.8 (1) |
| rs | uniform | 1 | 5 |
| Nan | Log uniform | 100 | 10,000 |
| Nas | Log uniform | 100 | 10,000 |
| Ncn | Log uniform | 1000 | 100,000 |
| Ncs | Log uniform | 1000 | 100,000 |
| mut | Log uniform | 1E-7** | 1E-6** |

* only in the *admixture* and *admixture_tot* models. *Admixture_tot* is within brackets

**Supplementary Table S12**. Prior distribution associated to models’ parameters. . P is the proportion of Sardinian lineages coming from Neolithic Europe, rs is the extent of population reduction due to the bottleneck of the first colonization of Sardinia, Nan is the ancient effective population size of Neolithic Europe, Nas is the ancient Sardinian effective population size, Ncn is the current European effective population size, Ncs is the current Sardinian effective population size and mut is the mutation rate per nucleotide per year.

Supplementary Table S13 – Dataset of the samples considered in the ABC analysis

| Sample | Time period | Country | Reference |
| --- | --- | --- | --- |
| OGL1 | Modern | Sardinia (Italy) | 22 |
| OGL2 | Modern | Sardinia (Italy) | 22 |
| OGL3 | Modern | Sardinia (Italy) | 22 |
| OGL4 | Modern | Sardinia (Italy) | 22 |
| OGL5 | Modern | Sardinia (Italy) | 22 |
| OGL6 | Modern | Sardinia (Italy) | 22 |
| OGL7 | Modern | Sardinia (Italy) | 22 |
| OGL8 | Modern | Sardinia (Italy) | 22 |
| OGL9 | Modern | Sardinia (Italy) | 22 |
| OGL10 | Modern | Sardinia (Italy) | 22 |
| OGL11 | Modern | Sardinia (Italy) | 22 |
| OGL12 | Modern | Sardinia (Italy) | 22 |
| OGL13 | Modern | Sardinia (Italy) | 22 |
| OGL14 | Modern | Sardinia (Italy) | 22 |
| OGL15 | Modern | Sardinia (Italy) | 22 |
| OGL16 | Modern | Sardinia (Italy) | 22 |
| OGL17 | Modern | Sardinia (Italy) | 22 |
| OGL18 | Modern | Sardinia (Italy) | 22 |
| OGL19 | Modern | Sardinia (Italy) | 22 |
| OGL20 | Modern | Sardinia (Italy) | 22 |
| OGL21 | Modern | Sardinia (Italy) | 22 |
| OGL22 | Modern | Sardinia (Italy) | 22 |
| OGL23 | Modern | Sardinia (Italy) | 22 |
| OGL24 | Modern | Sardinia (Italy) | 22 |
| OGL25 | Modern | Sardinia (Italy) | 22 |
| OGL26 | Modern | Sardinia (Italy) | 22 |
| OGL27 | Modern | Sardinia (Italy) | 22 |
| OGL28 | Modern | Sardinia (Italy) | 22 |
| OGL29 | Modern | Sardinia (Italy) | 22 |
| OGL30 | Modern | Sardinia (Italy) | 22 |
| OGL31 | Modern | Sardinia (Italy) | 22 |
| OGL32 | Modern | Sardinia (Italy) | 22 |
| OGL33 | Modern | Sardinia (Italy) | 22 |
| OGL34 | Modern | Sardinia (Italy) | 22 |
| OGL35 | Modern | Sardinia (Italy) | 22 |
| OGL36 | Modern | Sardinia (Italy) | 22 |
| OGL37 | Modern | Sardinia (Italy) | 22 |
| OGL38 | Modern | Sardinia (Italy) | 22 |
| OGL39 | Modern | Sardinia (Italy) | 22 |
| OGL40 | Modern | Sardinia (Italy) | 22 |
| OGL41 | Modern | Sardinia (Italy) | 22 |
| OGL42 | Modern | Sardinia (Italy) | 22 |
| OGL43 | Modern | Sardinia (Italy) | 22 |
| OGL44 | Modern | Sardinia (Italy) | 22 |
| OGL45 | Modern | Sardinia (Italy) | 22 |
| OGL46 | Modern | Sardinia (Italy) | 22 |
| OGL47 | Modern | Sardinia (Italy) | 22 |
| OGL48 | Modern | Sardinia (Italy) | 22 |
| OGL49 | Modern | Sardinia (Italy) | 22 |
| OGL50 | Modern | Sardinia (Italy) | 22 |
| OGL51 | Modern | Sardinia (Italy) | 22 |
| OGL52 | Modern | Sardinia (Italy) | 22 |
| OGL53 | Modern | Sardinia (Italy) | 22 |
| OGL54 | Modern | Sardinia (Italy) | 22 |
|  |  |  |  |

|  | OGL55 | Modern | Sardinia (Italy) | 22 |
| --- | --- | --- | --- | --- |
|  | OGL56 | Modern | Sardinia (Italy) | 22 |
|  | OGL57 | Modern | Sardinia (Italy) | 22 |
|  | OGL58 | Modern | Sardinia (Italy) | 22 |
|  | OGL59 | Modern | Sardinia (Italy) | 22 |
|  | OGL60 | Modern | Sardinia (Italy) | 22 |
|  | OGL61 | Modern | Sardinia (Italy) | 22 |
|  | OGL62 | Modern | Sardinia (Italy) | 22 |
|  | OGL63 | Modern | Sardinia (Italy) | 22 |
|  | I0019 | Early Neolithic | Germany | 49 |
|  | I0020 | Early Neolithic | Germany | 49 |
|  | I0021 | Early Neolithic | Germany | 49 |
|  | I0022 | Early Neolithic | Germany | 49 |
|  | I0023 | Early Neolithic | Germany | 49 |
|  | I0024 | Early Neolithic | Germany | 49 |
|  | I0025 | Early Neolithic | Germany | 49 |
|  | I0026 | Early Neolithic | Germany | 49 |
|  | I0027 | Early Neolithic | Germany | 49 |
|  | I0046 | Early Neolithic | Germany | 49 |
|  | I0048 | Early Neolithic | Germany | 49 |
|  | I0054 | Early Neolithic | Germany | 49 |
|  | I0056 | Early Neolithic | Germany | 49 |
|  | I0057 | Early Neolithic | Germany | 49 |
|  | I0100 | Early Neolithic | Germany | 49 |
|  | I0101 | Early Neolithic | Germany | 49 |
|  | I0102 | Early Neolithic | Germany | 49 |
|  | I0162 | Early Neolithic | Germany | 49 |
|  | I0163 | Early Neolithic | Germany | 49 |
|  | I0165 | Early Neolithic | Germany | 49 |
|  | I0166 | Early Neolithic | Germany | 49 |
|  | I0170 | Early Neolithic | Germany | 49 |
|  | I0659 | Early Neolithic | Germany | 49 |
|  | I0795 | Early Neolithic | Germany | 49 |
|  | I0796 | Early Neolithic | Germany | 49 |
|  | I0797 | Early Neolithic | Germany | 49 |
|  | I0820 | Early Neolithic | Germany | 49 |
|  | I0821 | Early Neolithic | Germany | 49 |
|  | I0172 | Middle Neolithic | Germany | 49 |
|  | I0212 | Middle Neolithic | Germany | 49 |
|  | I0548 | Middle Neolithic | Germany | 49 |
|  | I0549 | Middle Neolithic | Germany | 49 |
|  | I0551 | Middle Neolithic | Germany | 49 |
|  | I0552 | Middle Neolithic | Germany | 49 |
|  | I0554 | Middle Neolithic | Germany | 49 |
|  | I0556 | Middle Neolithic | Germany | 49 |
|  | I0557 | Middle Neolithic | Germany | 49 |
|  | I0559 | Middle Neolithic | Germany | 49 |
|  | I0560 | Middle Neolithic | Germany | 49 |
|  | I0798 | Middle Neolithic | Germany | 49 |
|  | I0799 | Middle Neolithic | Germany | 49 |
|  | I0800 | Middle Neolithic | Germany | 49 |
|  | I0802 | Middle Neolithic | Germany | 49 |
|  | I0807 | Middle Neolithic | Germany | 49 |
|  | I0808 | Middle Neolithic | Germany | 49 |
|  | I0822 | Middle Neolithic | Germany | 49 |
|  |  |  |  |  |

**Supplementary Table S13.** Dataset of the samples considered in the ABC analysis.

**References**

1. Atzeni,. E. Notiziario - Sardegna: Provincia di Cagliari. Riparo sotto roccia di «Su Carròppu» (Sirri-Carbonia). *Rivista di Scienze Preistoriche* **XXXII**, 357-358 (1978).
2. Gassin B. & Lugliè, C. La preistoria el aprotostoria della Sardegna. *Atti della XLIV R.S. IIPP* **II**, 485-493 (2012).
3. Lugliè. C. The Su Carroppu rock shelter within the process of Neolithization of Sardinia. In: *Transitions* *en Méditerranée, ou comment des chasseurs devinrent agriculteurs* (eds) (2014).
4. Dabney, J., *et al*. Complete mitochondrial genome sequence of a Middle Pleistocene cave bear reconstructed from ultra-short DNA fragments. *Proc Natl Acad Sci USA* **110**, 15758-63 (2013)..
5. Meyer, M. & Kircher, M. Illumina sequencing library preparation for highly multiplexed target capture and sequencing. *Cold Spring Harb Protoc* **2010**, pdb prot5448 (2010).
6. Schuenemann, V.J. *et al.* Genome-wide comparison of medieval and modern Mycobacterium leprae. *Science* **341**, 179-183 (2013).
7. Maricic, T., Whitten, M. & Paabo S. Multiplexed DNA sequence capture of mitochondrial genomes using PCR products. *PLoS One* **5**, e14004 (2010).
8. J. St. John. SeqPrep. eds (2011).
9. Li, H. & Durbin, R. Fast and accurate short read alignment with Burrows-Wheeler transform. *Bioinformatics* **25**, 1754-1760 (2009).
10. Schubert, M. *et al.* Improving ancient DNA read mapping against modern reference genomes. *BMC* *Genomics* **13**, 178 (2012).
11. Li, H. *et al.* The Sequence Alignment/Map format and SAMtools. *Bioinformatics* **25**, 2078-2079 (2009).
12. McKenna, A. *et al.* The Genome Analysis Toolkit: a MapReduce framework for analyzing next-generation DNA sequencing data. *Genome Res* **20**, 1297-1303 (2010).
13. Milne, I. *et al.* Tablet--next generation sequence assembly visualization. *Bioinformatics* **26**, 401-402 (2010).
14. van Oven, M. & Kayser, M. Updated Comprehensive Phylogenetic Tree of Global Human Mitochondrial DNA Variation. *Hum Mutat* **30**, 386-394 (2009).
15. Kloss-Brandstatter, A. *et al.* HaploGrep: a fast and reliable algorithm for automatic classification of mitochondrial DNA haplogroups. *Hum Mutat* **32**, 25-32 (2011).
16. Hofreiter, M., Jaenicke, V., Serre, D., Haeseler, A. & Paabo, S. DNA sequences from multiple amplifications reveal artifacts induced by cytosine deamination in ancient DNA. *Nucleic Acids Res* **29**, 4793-4799 (2001).
17. Sawyer, S., Krause, J., Guschanski, K., Savolainen, V. & Paabo S. Temporal patterns of nucleotide misincorporations and DNA fragmentation in ancient DNA. *PLoS One* **7**, e34131 (2012).
18. Briggs, A.W. *et al.* Patterns of damage in genomic DNA sequences from a Neandertal. *Proc Natl Acad* *Sci U S A* **104**, 14616-14621 (2007).
19. Jonsson, H., Ginolhac, A., Schubert, M., Johnson, P.L. & Orlando L. mapDamage2.0: fast approximate Bayesian estimates of ancient DNA damage parameters. *Bioinformatics* **29**, 1682-1684 (2013).
20. Fu, Q. *et al.* A revised timescale for human evolution based on ancient mitochondrial genomes. *Curr* *Biol* **23**, 553-559 (2013).
21. Haak, W. *et al.* Ancient DNA from the first European farmers in 7500-year-old Neolithic sites. *Science* **310**, 1016-1018 (2005).
22. Fraumene, C., Petretto, E., Angius, A. & Pirastu, M. Striking differentiation of sub-populations within a genetically homogeneous isolate (Ogliastra) in Sardinia as revealed by mtDNA analysis. *Hum Genet* **114**, 1-10 (2003).
23. Edgar, R.C. MUSCLE: a multiple sequence alignment method with reduced time and space complexity.*BMC Bioinformatics* **5**, 113 (2004).
24. Spoor, F. The human fossils from Corbeddu Cave, Sardinia: a reappraisal. In: *Elephants have a snorkel!* (eds Reumer JWFDVs, J. St. John). Deinsea (1999).
25. Excoffier, L. & Lischer, H.E. Arlequin suite ver 3.5: a new series of programs to perform population genetics analyses under Linux and Windows. *Mol Ecol Resour* **10**, 564-567 (2010).
26. Wegmann, D., Leuenberger, C., Neuenschwander, S. & Excoffier, L. ABCtoolbox: a versatile toolkit for approximate Bayesian computations. *BMC Bioinformatics* **11**, 116 (2010).
27. Posth, C. *et al.* Pleistocene Mitochondrial Genomes Suggest a Single Major Dispersal of Non-Africans and a Late Glacial Population Turnover in Europe. *Curr Biol* **26**, 827-833 (2016).
28. Jones, E.R. *et al.* Upper Palaeolithic genomes reveal deep roots of modern Eurasians. *Nat Commun* **6**, 8912 (2015).
29. Bollongino, R. *et al.* 2000 years of parallel societies in Stone Age Central Europe. *Science* **342**, 479-481 (2013).
30. Benazzi, S. *et al.* Archaeology. The makers of the Protoaurignacian and implications for Neandertal extinction. *Science* **348**, 793-796 (2015).
31. Krause, J. *et al.* The complete mitochondrial DNA genome of an unknown hominin from southern Siberia. *Nature* **464**, 894-897 (2010).
32. Sanchez-Quinto, F. *et al.* Genomic affinities of two 7,000-year-old Iberian hunter-gatherers. *Curr Biol* **22**, 1494-1499 (2012).
33. Lazaridis, I. *et al.* Ancient human genomes suggest three ancestral populations for present-day Europeans. *Nature* **513**, 409-413 (2014).
34. Raghavan, M. *et al.* Upper Palaeolithic Siberian genome reveals dual ancestry of Native Americans. *Nature* **505**, 87-91 (2014).
35. Fu, Q. *et al.* Genome sequence of a 45,000-year-old modern human from western Siberia. *Nature* **514**, 445-449 (2014).
36. Pala, M. *et al.* Mitochondrial DNA signals of late glacial recolonization of Europe from near eastern refugia. *Am J Hum Genet* **90**, 915-924 (2012).
37. Greenspan, B. FTDNA Direct submission. *Genebank* (2010).
38. Raule, N. *et al*. The co-occurrence of mtDNA mutations on different oxidative phosphorylation subunits, not detected by haplogroup analysis , affects human longevity and is population specific. *Aging Cell* **3**, 401-407 (2014).
39. Greenspan, B. FTDNA Direct submission. *Genebank* (2011).
40. Moilanen, J.S. *et al*. Lineage-Specific Selection in Human mtDNA: Lack of Polymorphisms in a segment of MTND5 Gene in Haplogroup J. *Mol Biol Evol* **20**, 2132-2142 (2003).
41. Greenspan, B. FTDNA Direct submission. *Genebank* (2008).
42. Fraumene, C. *et al*. High resolution analysis and phylogenetic network construction using complete mtDNA sequences in sardinian genetic isolates. *Mol Biol Evol* **23**, 2101-2111 (2006).
43. Hartmann, A. *et al*. Validation of microarray-based resequencing of 93 worldwide mitochondrial genomes. *Hum Mutat* **30**, 115-122 (2009).
44. Lander, E.S. *et al*. Initial sequencing and analysis of the human genome. *Nature* **409**, 860-921 (2001).
45. Gomez-Carballa, A. *et al*. Evolutionary analyses of entire genomes do not support the association of mtDNA mutations with Ras/MAPK pathway syndromes. *PLoS One* **6**, e18348 (2011).
46. Cassidy, L.M. *et al*. Neolithic and Bronze Age migration to Ireland and establishment of the insular Atlantic genome. *Proc Natl Acad Sci USA* **113**, 368-373 (2016).
47. Allentoft, M.E. *et al*. Population genomics of Bronze Age Eurasia. *Nature* **522**, 167-172 (2015).
48. Mathieson, I. *et al*. Genome-wide patterns of selection in 230 ancient Eurasians. *Nature* **528**, 499-503 (2015).
49. Haak, W. *et al*. Massive migration from the steppe was a source for Indo-European languages in Europe. *Nature* **522**, 207-211 (2015).
